# Biogenesis, inheritance, and 3D ultrastructure of the microsporidian mitosome

**DOI:** 10.26508/lsa.202201635

**Published:** 2023-10-30

**Authors:** Christian Hacker, Kacper Sendra, Priyanka Keisham, Teodora Filipescu, James Lucocq, Fatemeh Salimi, Sophie Ferguson, David Bhella, Stuart A MacNeill, Martin Embley, John Lucocq

**Affiliations:** 1 School of Medicine, University of St Andrews , St Andrews, UK; 2 Biosciences Institute, The Medical School, Catherine Cookson Building, Newcastle University, Newcastle upon Tyne, UK; 3 MRC-University of Glasgow Centre for Virus Research, Glasgow, UK; 4 Department of Surgery, Dundee Medical School Ninewells Hospital, Dundee, UK; 5 School of Biology, University of St Andrews , St Andrews, UK; 6 Biosciences Institute, Centre for Bacterial Cell Biology, Baddiley-Clark Building, Newcastle University, Newcastle upon Tyne, UK

## Abstract

During the reductive evolution of obligate intracellular parasites called microsporidia, a tiny remnant mitochondrion (mitosome) lost its typical cristae, organellar genome, and most canonical functions. Here, we combine electron tomography, stereology, immunofluorescence microscopy, and bioinformatics to characterise mechanisms of growth, division, and inheritance of this minimal mitochondrion in two microsporidia species (grown within a mammalian RK13 culture-cell host). Mitosomes of *Encephalitozoon cuniculi* (2–12/cell) and *Trachipleistophora hominis* (14–18/nucleus) displayed incremental/non-phasic growth and division and were closely associated with an organelle identified as equivalent to the fungal microtubule-organising centre (microsporidian spindle pole body; mSPB). The mitosome–mSPB association was resistant to treatment with microtubule-depolymerising drugs nocodazole and albendazole. Dynamin inhibitors (dynasore and Mdivi-1) arrested mitosome division but not growth, whereas bioinformatics revealed putative dynamins Drp-1 and Vps-1, of which, Vps-1 rescued mitochondrial constriction in dynamin-deficient yeast (*Schizosaccharomyces pombe*). Thus, microsporidian mitosomes undergo incremental growth and dynamin-mediated division and are maintained through ordered inheritance, likely mediated via binding to the microsporidian centrosome (mSPB).

## Abstract (precis)

Quantitative electron and light microscopy combined with bioinformatics reveal that microsporidian mitosomes grow and divide incrementally and are inherited at the microsporidian centrosome (spindle pole body), whereas mitochondrial dynamins participate in mitosome division.

## Introduction

A fundamental question in cell biology is how organelles are maintained in growing cell populations (Warren & Wickner, 1996; Mullock & Luzio, 2000; Giacomello & Pellegrini, 2016). In the case of particulate organelles, this occurs by proportionate inheritance at cell division and appropriate growth, and multiplication (division) in the next interphase (Warren & Wickner, 1996). Inheritance can be stochastic, where abundant and widely distributed organelles (e.g., proteasomes or ribosomes) are divided equally by cytokinesis. Alternatively, inheritance can be ordered, whereby rare, essential organelles such as chromosomes are inherited via association with cell structures such as the cytoskeleton. For single or interconnected organelles, fission can yield either two fragments that are distributed to the daughter cells by ordered inheritance or produce many more copies that are then inherited probabilistically. When organelles are already present in multiple copies, dispersal in the cytoplasm ensures proportionate delivery into daughter cells.

Mitochondria are essential, semiautonomous, particulate organelles (Ali & Nozaki, 2013; Braymer & Lill, 2017; Roger et al, 2017), with critical roles in energy production (oxidative phosphorylation), calcium regulation, apoptosis, and iron sulphur cluster assembly (Freibert et al, 2017). Because mitochondria cannot be made de novo (Ploumi et al, 2017), eukaryotes have evolved reliable mechanisms for their inheritance, growth, and multiplication (Mishra & Chan, 2014). In mammalian cells, early studies (Posakony et al, 1977) indicated that the mitochondrial compartment first expands during interphase and then undergoes fragmentation and dispersal in mitosis to be inherited by probabilistic stochastic mechanisms (Posakony et al, 1977; Taguchi et al, 2007; Kianian & Kianian, 2014; Mishra & Chan, 2014; Jongsma et al, 2015; Lawrence et al, 2016). By comparison in yeast (and other eukaryotes [Melo et al, 2000; Ogbadoyi et al, 2003]), where mitochondria are less abundant, inheritance proceeds by precise positioning before delivery into the daughter cells. In the budding yeast *Saccharomyces cerevisiae*, mitochondria become located at the emergent daughter cell through cytoskeletal tethers (Knoblach & Rachubinski, 2015; Pernice et al, 2018), whereas in the fission yeast *Schizosaccharomyces pombe*, a subpopulation associates with the centrosome (spindle pole body [SPB]; Yaffe et al, 2003; Krüger & Tolić-Nørrelykke, 2008), ensuring inheritance for the next growth cycle (Newton & Fangman, 1975). Therefore, current data suggest that when the mitochondrial abundance is low, ordered inheritance predominates, whereas when mitochondria are numerous/abundant, ordered inheritance is relaxed and stochastic strategies predominate.

Mitochondrial number is determined by the balance between fusion and fission, a process that is responsive to cues from cell cycle and cellular stress (Nunnari et al, 2012; Park & Cho, 2012; Lopez-Mejia & Fajas, 2015). At the molecular level, fission/fusion has a well-characterised machinery comprising a number of large GTPases (Park & Cho, 2012; Lackner et al, 2013; Lawrence et al, 2016; Wai & Langer, 2016; Tilokani et al, 2018). Fusion at the outer membrane involves mitofusins 1 and 2, and fusion at the inner membrane uses OPA1 (Chandhok et al, 2018). Fission is driven by Drp1 and dynamin 2, that work in sequence to first constrict (Drp1) and execute scission (dynamin 2) (Kraus & Ryan, 2017; Tilokani et al, 2018). Fission is also assisted by a series of adaptors (Yu et al, 2020) and by actin and myosin, which cooperate with the ER in initiating constriction at future sites of scission (Chakrabarti et al, 2018). During mitosis, Drp1 is phosphorylated by CDK1/cyclin (Ishihara et al, 2013) to drive mitochondrial fission (Taguchi et al, 2007). Drp1 is then degraded during mitosis (Horn et al, 2011; Lopez-Mejia & Fajas, 2015), which contributes to restoration of the interphase mitochondrial network.

In this report, we present data on the mitochondrial biogenesis and inheritance of a specialised mitochondrion, the mitosome. Mitosomes are evolutionarily stripped-down mitochondria that lack a genome and have secondarily lost canonical functions (Makiuchi & Nozaki, 2014) as adaptations to a parasitic lifestyle. The retention of mitochondrial-related organelles in most eukaryotes likely reflects the functional and essential importance of iron sulphur cluster assembly (Paul & Lill, 2015; Pyrih et al, 2016; Freibert et al, 2017). Mitosomes are tiny (measuring 50–150 nm) and retain a double membrane but remain poorly characterised morphologically. Patterns and mechanisms of mitosome biogenesis and inheritance are also poorly understood. To date, studies on mitosome life cycle are limited to studies of the parasite *Giardia intestinalis* using light microscopy, in which mitosomes appear to undergo a mixture of ordered and random inheritance, with flagella playing a key role in conserving transfer to daughter organelles (Regoes et al, 2005; Tůmová et al, 2021). Detailed elucidation and understanding of mitosome biogenesis, inheritance, and structure now require quantitative ultrastructural studies.

Here, we present a study of mitosome biogenesis in a prominent group of obligate intracellular parasites, called microsporidia. These organisms are widespread in the biosphere, causing significant disease in humans and economically important animals. They are characterised by markedly reduced genomes, metabolism, and organelles and supplement their metabolism by stealing molecules from the host (Tsaousis et al, 2008; Dean et al, 2014). Mitosomes of microsporidian *Trachipleistophora hominis* were previously demonstrated as a potential target of therapeutic intervention (Sendra et al, 2022). Although, because of their obligate intracellular lifestyle, microsporidians are not easily amenable to genetic modification and live cell fluorescence work, their mitosomes are prominent and of low abundance, making them ideal objects for studying mitochondrial biogenesis and inheritance using electron microscopy.

Electron tomography (ET) reveals microsporidian mitosomes as dumbbell-shaped organelles with a double-membraned hull while quantitative electron microscopy (stereology) shows the relatively few mitosomes of *Encephalitozoon cuniculi* grow and divide continuously during interphase, remaining tethered to centrosomes (microsporidian spindle pole bodies [mSPB]) throughout the cell cycle. In a second species, *T*. *hominis*, the more numerous mitosomes are largely dislocated from the mSPB during a syncytial phase but associate with the mSPB before cellularisation. Investigating duplication mechanisms, we detected two microsporidian dynamin homologues and found dynamin inhibitors reduce numbers and increase the size of mitosomes over a 2-h period. These microsporidian dynamins rescued mitochondrial constriction in a dynamin-deficient yeast.

Our data indicate that mitosome biogenesis in microsporidians combines non-phasic growth and ongoing dynamin-driven scission during interphase. The microsporidian centrosome appears to play a key role in distribution and inheritance of mitosomes, thereby ensuring their continued existence of these numerically rare organelles through the microsporidian life cycle.

## Results

### Morphological characterization of *E*. *cuniculi* mitosomes using electron tomography

We studied the distribution, morphology, and numbers of mitosomes through vegetative (meront; non-spore) stages of the life cycle of microsporidians *E. cuniculi* and *T. hominis*. *E. cuniculi* cells contain only a few mitosomes and are physically small (∼1 µm diameter) through their life cycle up to spore differentiation, and develop within a vacuole likely derived from host cell membranes (Katinka et al, 2001). Early meronts of *E. cuniculi* multiply in contact with the vacuole boundary, before detaching and differentiating (Rönnebäumer et al, 2008; Hacker et al, 2014). In contrast, the larger *T. hominis* harbours more numerous mitosomes, and becomes multinucleate before cellularisation and sporulation. A cell coat forms around early meronts and subsequently develops into the wall of the parasitophorous vacuole (Ferguson & Lucocq, 2019).

**Figure 1.**
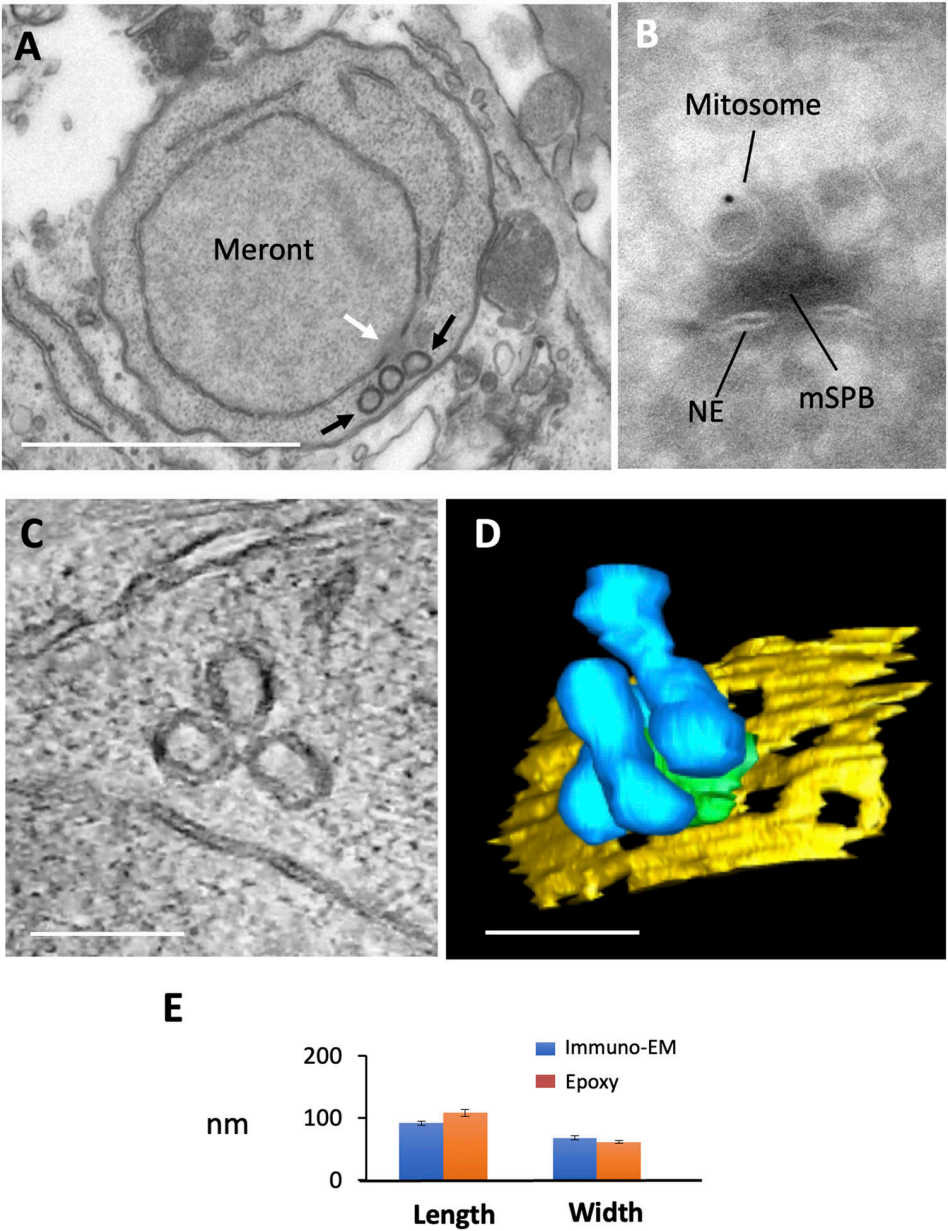
Double-membraned hsp70-positive structures in *E. cuniculi* have a dumbbell shape in 3D and associate with the spindle pole body. Conventional EM, immuno-EM, and ET structures of mitosomes in *E*. *cuniculi*-infecting monolayer cultures of RK13 cells. **(A)** Conventional epoxy resin section (∼40 nm thick) of a vegetative intracellular form (meront) reveals a group of three double-membraned structures (black arrows), situated close to the nucleus and associated with an amorphous electron dense structure (white arrow) in contact with the nuclear envelope (microsporidian spindle pole body [mSPB]). **(B)** Immunogold labelling for hsp70 in ultrathin cryosections reveals a double-membraned structure associated with the electron dense mSPB extending into a nuclear pore-like interval in the double-membraned nuclear envelope NE. **(C, D)** Electron tomography of mitosome groups at the mSPB in meronts. **(C)** slice view and (D) 3D reconstruction (see Fig S1 for Video 1 and Video 2). 200-nm-thick sections were imaged at 200 keV using a tilt series recorded at 1° intervals up to 50° tilt, before back-projection into 3D space (blue—mitosomes, yellow—nuclear envelope and green—mSPB). **(B, D, E)** Scale bars (A), 1 micron; (B) 50 nm; (D and E), 100 nm. **(E)** Comparison of profile length and width of double-membraned profiles in meronts identified by double membranes and hsp70 labelling. N = 3, error bars SEM.

**Figure S1.**
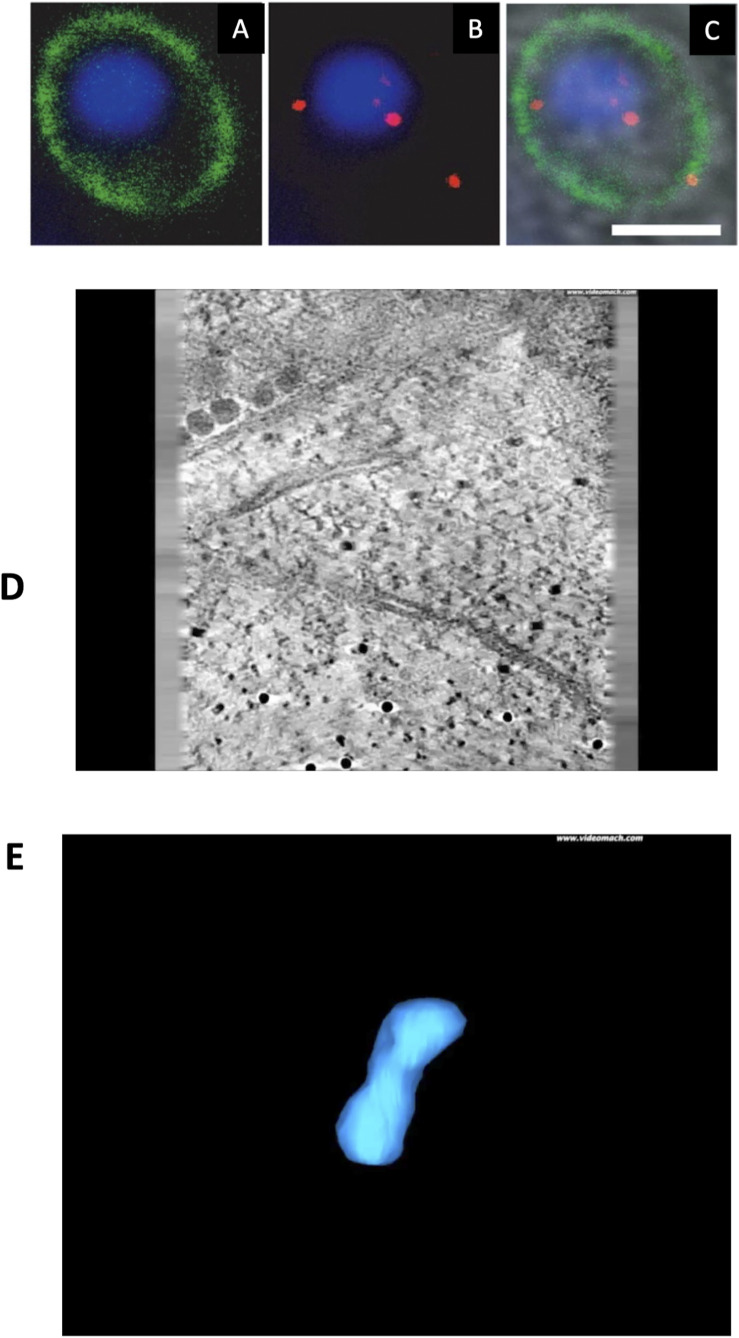
Immunofluorescence localization of mitosomes and 3D morphology from ET data in *E. cuniculi*. **(A, B, C)** Immunofluorescence localization of mitosomes using antibodies against hsp70 (red). **(A, B, C)** Single spots of stain close to the nucleus represent accumulations of mitosomes that are not resolved by this light microscopy method ((A), cell surface marker; (B) mitosome hsp70, and (C), merge). **(D)** Tomogram of a group of mitosomes from a single 200-nm-thick section. The two mitosomes to the left are complete. **(E)** Reconstruction of a group of mitosomes from a pair series of 200-nm-thick sections. Mitosomes are in blue, microsporidian spindle pole bodies in green, nuclear envelope in yellow, and ER in red.

To identify *E. cuniculi* mitosomes from structural criteria alone, thawed cryosections of *E. cuniculi*-infected host cells were immunogold labelled with antibodies against the specific mitosome marker Hsp70 (Fig 1A–E; IF in Fig S1A–C; [Williams et al, 2002; Tovar et al, 2003]). Labelled structures had a double-membrane hull (50–60 nm × ca. 150 nm) and similar structures were observed in epoxy resin sections (Fig 1C). Mitosomes were found closely associated with an amorphous structure located on the cytoplasmic aspect of the nuclear membrane, resembling the SPB in yeast (mSPB; see Fig 1A and B). Mitosome structure in *T. hominis* has been validated previously using immuno-EM (Williams et al, 2002).

Video 1 Tomogram of a group of mitosomes from a single 200-nm-thick section.

Video 2 Reconstruction of a group of mitosomes from a pair series of 200-nm-thick sections.

To reveal the detailed 3D-ultrastructure of the *E. cuniculi* mitosome, 200-nm-thick epoxy sections were subjected to ET (Figs 1C and D and S1D and E). Tomograms revealed that mitosomes lack demonstrable outer-inner membrane contact sites and possess hemi-spherical ends connected by a thinner “waist.” The mitosome matrix lacked discernible electron dense granules, fibrils or internal membranes. In reconstructions (Figs 1D and S1E), most mitosomes were in close contact with the homogeneous mSPB matrix Fig S1E; also previously referred to as the spindle pole plaque (Sacchi et al, 2007; Vavra & Lukes, 2013). By ET in epoxy resin mSPBs appeared homogeneous, although internal layering was visible after embedding in methacrylate/acrylate resin Lowicryl HM20 using progressively lowered temperature protocols (not shown).

In a wide range of eukaryotic cells, mitochondria make specialised contacts with other organelles including ER (Kornmann, 2013; Giacomello & Pellegrini, 2016). Mitochondria–ER contact zones exchange lipids and control early phases of mitochondrial scission (Friedman, et al, 2011; Giacomello & Pellegrini, 2016). In our conventional TEM experiments, *E. cuniculi* mitosomes were found in close association with ER (Fig S1D, coloured red), with 24 out of 36 identified groups of mitosomes forming at least one contact with ER; with 22% of 134 individual mitosomes making contact. Specialised structures such as increased electron density at the contact sites or wrapping of ER or juxtaposition of other organelles to the “waist” region of mitosomes often involved in scission of mitochondria in other organisms (Friedman et al, 2011; Tábara et al, 2021), were not identified.

### The electron dense plaque of microsporidians is homologous to the SPB of fungi

**Figure 2.**
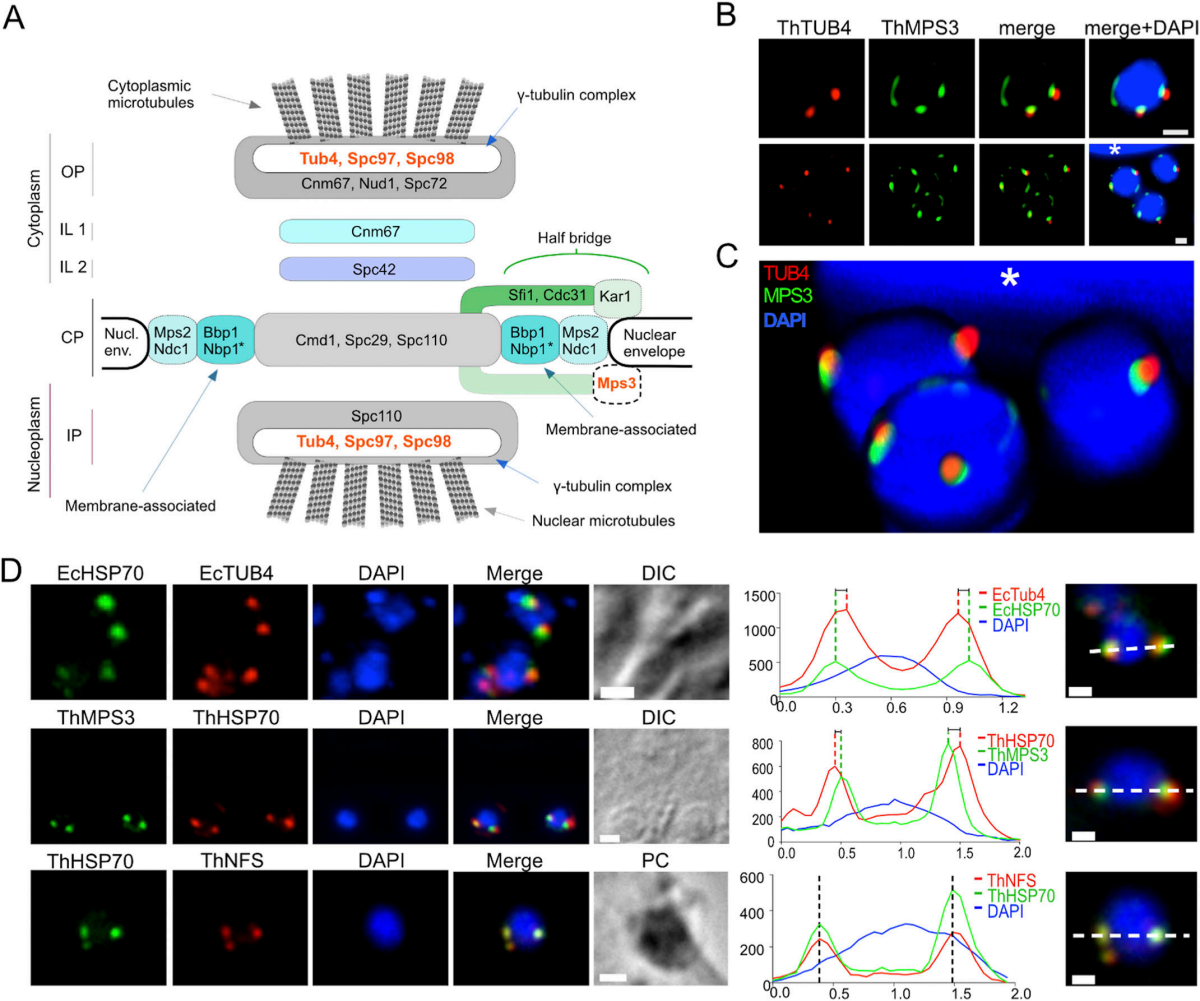
Characterisation of the microsporidian spindle pole body. **(A)** Model of *S. cerevisiae* spindle pole body (SPB) depicting relative position of SPB-protein components. Homologues of 4 out of the 21 *S. cerevisiae* SPB proteins were identified in microsporidian genomes (orange): the γ-tubulin complex components (TUB4, SPC97, and SPC98), and a nuclear membrane protein MPS3. **(B)** Cells of the microsporidian parasite *T. hominis* grown inside a monolayer of rabbit kidney RK13 host cells were labelled with specific antibodies against *T. hominis* homologues of γ-tubulin (TUB4, red) and MPS3 (green). Similar labelling patterns were observed in single nucleate early meront (top row) and multinucleate late meronts (bottom row). Images are maximum intensity Z-projections of deconvolved confocal Z-stacks (Fig S3). **(B, C)** A 3D visualisation of the 3 nuclei of the multinucleate *T. hominis* meront (B) bottom row, and Fig S3) inside a host cell. A small visible section of a large nucleus of the infected host cell was indicated at the top of the image (asterisk). **(D)** Localisation of mitosomal markers (HSP70 and NFS) and SPB homologues (TUB4 and MPS3) in *E. cuniculi* (Ec) and *T*. *hominis* (Th) meronts respectively. Closely associated signals for microsporidian spindle pole bodies and mitosome markers are shown in the intensity plots (arbitrary units). Bars 0.5 µm.

To test whether the electron dense plaque of microsporidians is homologous to the fungal SPB, we searched for homologues of known fungal SPB proteins in available microsporidian genomes. We identified four homologues out of the 21 *S. cerevisiae* SPB proteins: namely the γ-tubulin complex components (TUB4, SPC97, and SPC98) and a nuclear membrane protein MPS3 (Fig 2A). Microsporidian genomes have undergone extreme reduction and divergence during the adaptation to their obligate intracellular parasitic lifestyle. Evolutionary retention of the homologues of γ-tubulin and MPS3 most likely reflect the important roles played by these proteins. Our inability to detect other components of the SPB suggest either they are too divergent to be detected using even the most sensitive bioinformatics tools, or they have been lost during reductive evolution of microsporidia.

**Figure S2.**
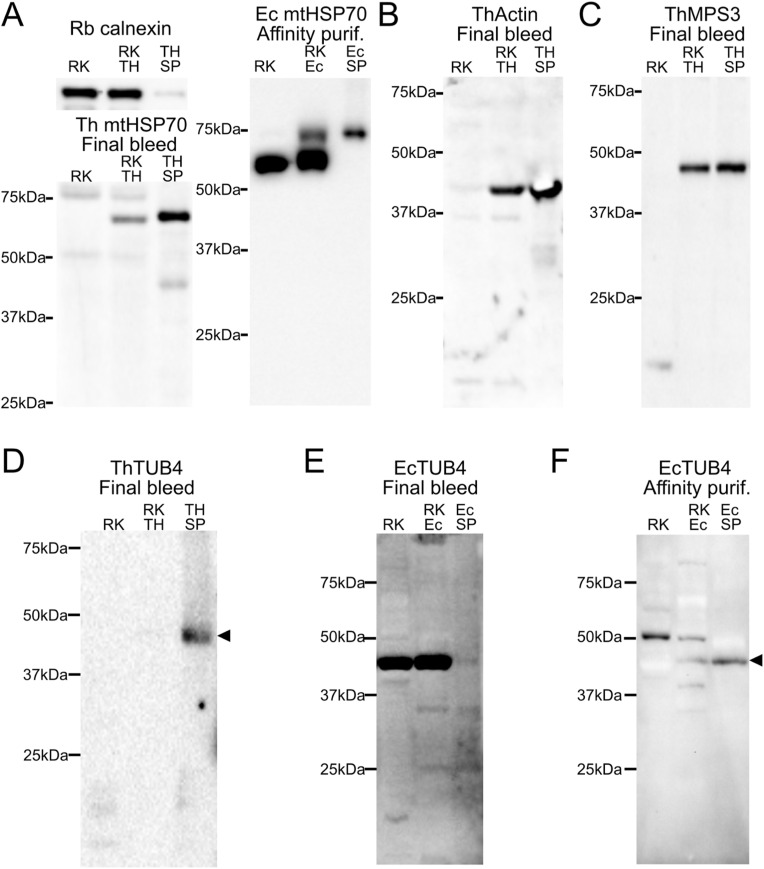
Characterisation of antibodies against microsporidian spindle pole bodies components by immunoblotting. **(A)** Control immunoblotting experiments with previously characterised specific antibodies against host and parasite proteins. Top panel—immunoblotting of *T. hominis* (Th) mitochondrial HSP70 (mtHSP70), rabbit (Rb) calnexin, Bottom panel—immunoblotting of *E. cuniculi* (Ec) mitochondrial HSP70 (mtHSP70). Samples were RK13 (RK), RK13 infected with *T. hominis* (RK Th) and purified spores from *T. hominis* infected RK13 (THSP), or RK13 infected with *E. cuniculi* (RK Ec) and purified spores from *E. cuniculi* infected RK13 (EcSP). **(B, C)** Final bleeds from rats immunised with *Th*Actin (B) or ThMPS3 (C) detected specific bands only in samples containing parasite proteins (RHTh, and THSP) but not in the control extracts containing only the host proteins (RK). **(D)** Final bleed from rabbit immunised with *Th*TUB4 detected a specific band (black arrowhead) mainly in protein extracts from purified spores (THSP). **(E, F)** The parasite-specific bands (black arrowhead) were detected with anti-*Ec*TUB4 antibodies after affinity purification of the final bleed sera against purified protein used to immunise the animal (F), but not using the non-purified final-bleed sera (E) where the main detected band corresponded to a nonspecific cross-reaction with a host protein.

**Figure S3.**
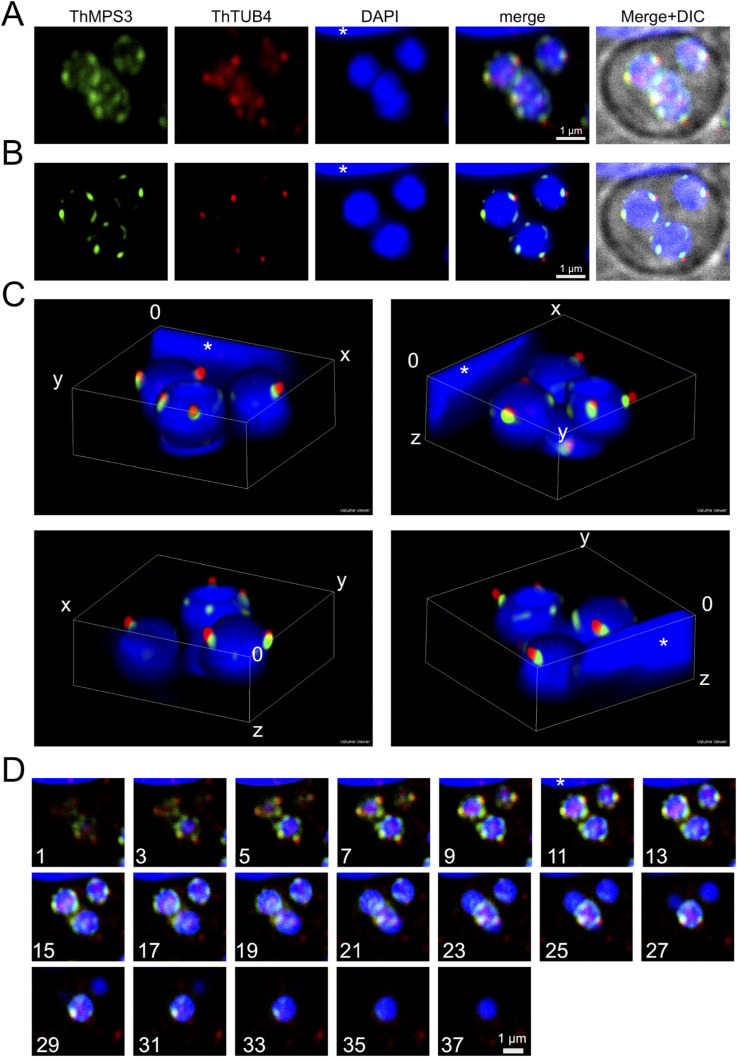
Localisation of SPB proteins MPS3 and TUB4 in a multinucleate *T. hominis* meront. A detailed analysis of the SBPs inside the multinucleate *T. hominis* meront presented in the Fig 2B (bottom panel) and Fig 2C. **(A, D)** In the non-deconvolved images (A, D), low intensity dispersed red fluorescence detected with the antibodies against *T. hominis* γ-tubulin was observed inside the *T. hominis* nuclei, suggesting that the intranuclear MTOCs organisation may differ from that of cytosolic MTOCs usually observed as well-defined punctate signals within the SPBs at the outer periphery of DAPI-stained nuclei. **(A)** A maximum intensity projection of a Z-stack of LCSM images sampled through a four-nucleate *T. hominis* meront inside a cytosol of an infected RK-13 host cell. The parasite SPB components were labelled with the rabbit anti-ThTUB4 (red) and the rat anti-ThMPS3 (green) antibodies. **(A, B)** A maximum intensity projection of a subset of deconvolved images from the Z-stack of the multinucleate *T. hominis* meront from (A). **(D)** For clarity, the subset of images ((D); 1–15) was selected to present the SPBs from the top three nuclei and remove the bottom nucleus that overlaps in the x- and y-dimensions with two out of three top-layer nuclei. **(B)** The fluorescence images in (B) are the same as in the main Fig 2B (bottom panel). **(A, B, C)** 3D-rendering of the Z-stack of deconvolved images of the cell presented in (A, B), and in Fig 2B and C. Each image represents a 90° rotation of the rendering. **(A, D)** Individual images from the non-deconvolved Z-stack (A). Images were sampled at 125 nm intervals in the z-axis. For clarity, only every second image was displayed. Parasite nuclei and host nuclei (white asterisks) were labelled with DAPI (blue).

To investigate the mSPB–mitosome relationship, we cloned, expressed, and raised antibodies against, microsporidian TUB4 (γ−tubulin) from *E. cuniculi* and TUB4 and MPS3 from *T. hominis* (Figs S2A–F and 2B and C). Immunofluorescence in widefield (Fig 2D), confocal microscopy (Fig 2B and C), and super-resolution STED microscopy (Fig 6C) revealed punctate signals for both markers that were situated on either side of the interphase nucleus, overlapping partially with mitosomal HSP70. Confirming structural EM results, spatial analysis showed mSPB protein signals interpose between nuclear DNA and mitosomal HSP signals (Fig 2D). Interestingly, MPS3 signals did not overlap completely with TUB4 and were more closely related to the nuclear DAPI signal (Figs 2B and S3). Some MPS3 signals were elongated and separated from the TUB4 signal, consistent with diverse and previously described nuclear functions in yeast (Prasada Rao et al, 2021; Fig 2B).

### Growth and division patterns of mitosomes in *E. cuniculi*

Our quantitative EM analysis of mitosome biogenesis concentrated on proliferating vegetative forms (meronts) of *E. cuniculi* that are found as a homogeneous population in the periphery of parasite vacuoles and lack signs of differentiation into spores (polar tube and cell wall) (Bohne et al, 2011; Hacker et al, 2014). The aim was to use ultrastructural quantification to correlate mitosome size and number with cell growth (and therefore, cell cycle), but this presented two major methodological challenges.

**Figure 3.**
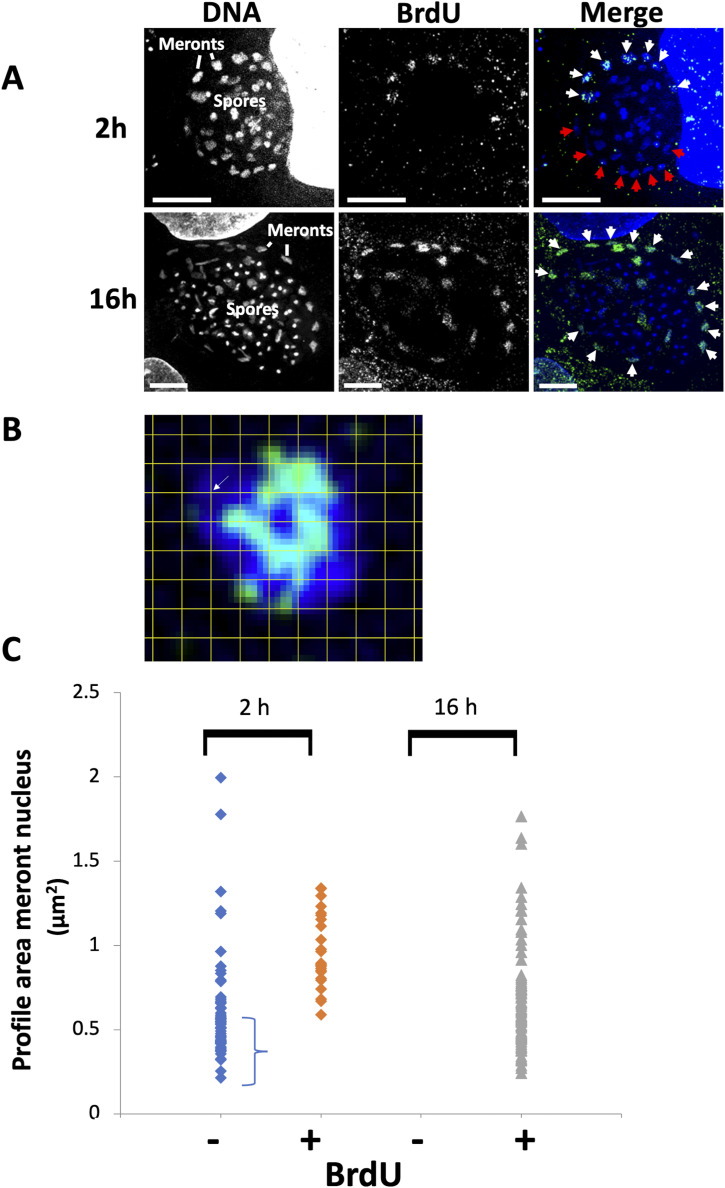
BrdU labelling identifies larger cells consistent with late cell cycle. BrdU analysis of *E*. *cuniculi* cell cycle in RK13 cells. After 2 or 16 h BrdU incubation, incorporation was detected using antibodies to BrdU and vegetative (meront) forms situated at the periphery of the parasite groups at the location of the vacuole membrane (not seen here) were analysed. **(A)** Qualitative analysis of BrdU labelling. At 2 h, groups of BrdU-positive (white arrows) and BrdU-negative cells (red arrows) are apparent, whereas at 16 h, all of the meronts incorporate label (white arrows). Some spores have incorporated BrdU and likely represent differentiated meronts (spores are unlikely to label because of their thick cell wall). **(B)** Stereological estimation of nuclear profile area. A square lattice grid with known spacing was positioned randomly over the nuclear profile. Points (represented by corners of the lattice, e.g., white arrow) were counted and area estimated from the number of points multiplied by the area associated with each point. **(C)** Correlation of BrdU labelling with meront nuclear size. Meronts were classified into BrdU positive and negative, and areas of the nuclear profiles assessed stereologically as described in (B), (Lucocq, 2008). At 2 h a population of cells with smaller nuclei are negative for BrdU (60/84) whereas after 16 h BrdU label, cells with smaller nuclei are all labelled (88/88). The results are consistent with cells with smaller nuclei being early in the cell cycle.

**Figure S4.**
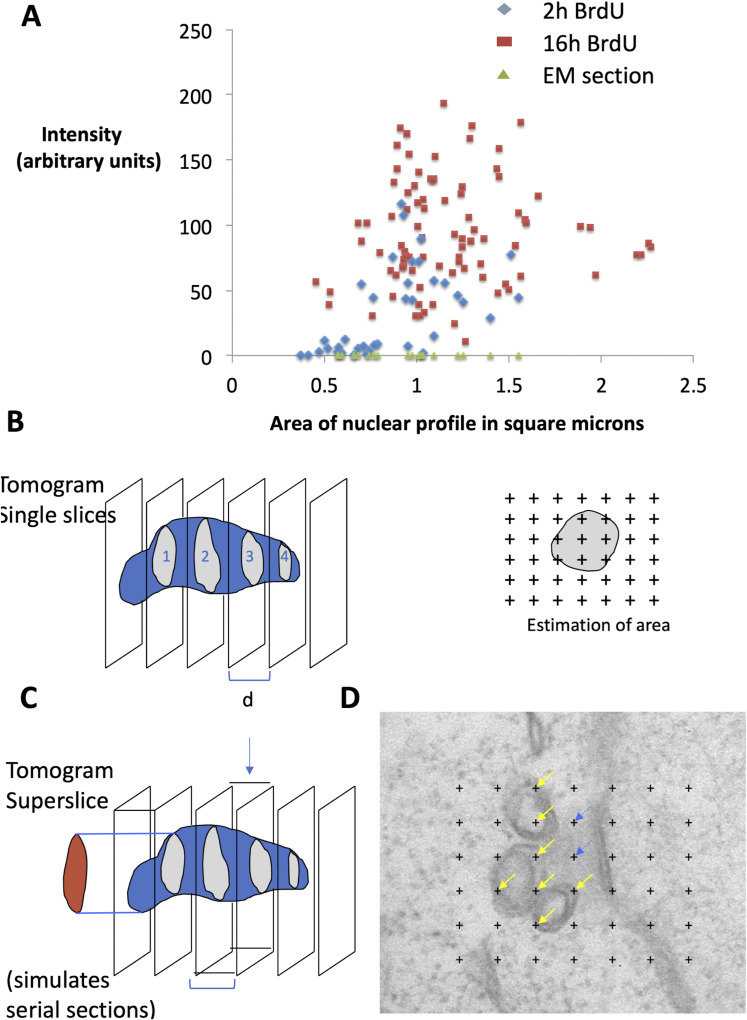
Methodological workup for quantitative electron microscopy. **(A)** Comparison between nuclear profile size in BrdU (light microscopy) and maximum projection of ER serial section images. Unlabelled cells with low or absent BrdU fluorescence are smaller and labelled cells larger after 2 h treatment, whereas nearly all cells become labelled after 16 h treatment. The nuclear profile sizes from the serial section electron microscopy sample used in this study span a size range across the major portion of the profile size distribution obtained from the meronts by BrdU light microscopy. **(B, C, D)** Correction for overprojection using tomographic slices assembled into superslices. **(B)** Volume is estimated using Cavalieri’s method using a randomly placed section stack of individual tomographic slices through a small object such as the mitosome (∼50 nm across). The slices generate profiles (grey) of which the areas can be estimated using point counting. Area = point hits over profile x area associated with each point. The sum of all estimated areas across all slice profiles (1, 2, 3, and 4) multiplied by the spacing between the slices (d) is an estimate of volume. The point counting procedure is illustrated on the example of slice profile 1 to the right of the slice array. **(C)** To simulate the conventional TEM section-thickness error, the individual tomographic slices are assembled into blocks of 43 nm thickness, and the volume of the same object is estimated from projections from these thicker slices (red). In the superslice left (arrow), the increase in size of the apparent area of the profile because of overprojection (red) is compared with the profile found in the tomographic slice (grey). In the real world of TEM microscopy, this error occurs because the real section has a thickness of 43 nm and is imaged as a projection in the electron microscope. In Cavalieri volume estimations, the overprojection areas from the superslices (conventional TEM thickness sections) can be compared with the volume obtained using much thinner tomographic slices (green). The ratio of the two volume estimations was used to calculate a correction factor (1/1.61). **(D)** illustrates the counting procedure on real images. The regular point lattice with known spacing is placed randomly over structures of interest. Point hits that lie over mitosome structures (yellow) and amorphous microsporidian spindle pole bodies structures (blue) are illustrated and used as described in part (B) above to generate volume estimates.

The first was to establish links between the cell cycle and cell size, so that we could infer cell cycle position from cell or nuclear volumes estimated using EM stereology. By correlating BrdU labelling with size determinations in light and electron microscopy (See Figs 3A–C and S4A), we found cells that labelled during a short pulse of BrdU (i.e., during S phase) contained the largest nuclei. By contrast, cell nuclei labelled during more extended labelling times were on average much smaller and included earlier cell cycle stages. Next, by comparing the sizes of nuclear profiles in light and electron microscopy (Fig S4A), it was possible for the nuclear size to be used as a yardstick for comparing cell cycle position with mitosome quantities (see below).

The second problem was to correct for an estimation bias that arises when EM stereology is used on conventional resin sections to quantify tiny structures such as mitosomes (Weibel & Paumgartner, 1978). When structures are similar in thickness to the slices used for stereology (epoxy resin sections are 40 nm and mitosomes are 50 nm), the structures “overproject” into the images and inflate the estimates (Fig S4B–D). In this case, the thinner slice thickness afforded by electron tomography can be used to develop a correction factor for both volume and number estimations (see the Materials and Methods section and Vanhecke et al [2007]).

With the link to cell cycle and the section thickness correction factor in place, mitosome quantities and nuclear size were compared with *E. cuniculi* meronts using stereology on serial ultrathin sections for TEM (Lucocq et al, 1989; Ferguson et al, 2017; Bohne et al, 2011; Hacker et al, 2014; see the Materials and Methods section). Data obtained from 18 cells revealed the average total mitosome volume per cell meront nucleus was 0.0038 μm^3^ (CE_18_, 10.4%) comprising 0.47% of cytoplasmic volume. The average number of mitosomes was 7.4 (CE_18_ 7.4%) per nucleus and the average volume 0.000628 μm^3^ (CE_18_ 7.6%). The smallest observed mitosome volume was 0.0001 μm^3^ which modelled as a sphere would measure 58 nm in diameter. The largest mitosome had a volume of 0.00025 μm^3^ which when modelled as a cylinder with hemispherical 50-nm diameter and ends would be 340 nm long.

**Figure 4.**
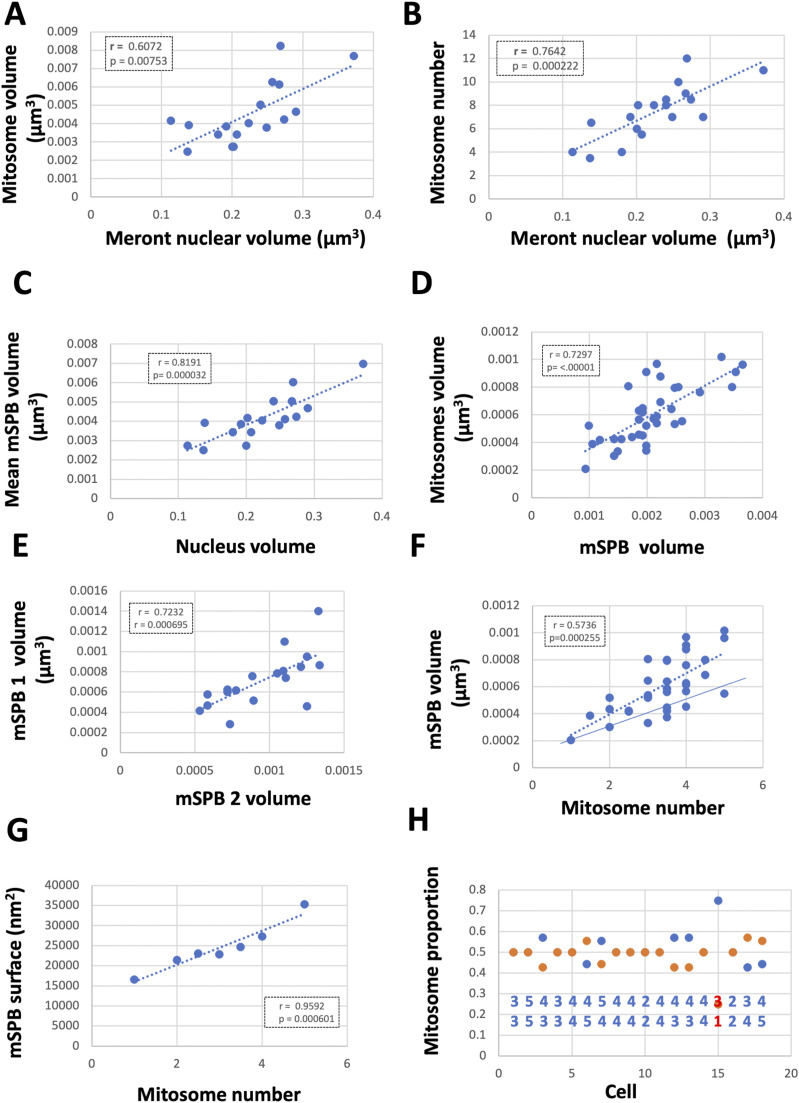
Mitosome growth and division in *E. cuniculi* meronts is non-phasic. **(A, B)** Mitosome volume (V mit) and number (N mit) increase with volume of parasite (meront) nucleus ((A, B), respectively). Relationships are consistent with gradual increments during the meront growth cycle (see also Table 1). **(C)** Mean volume of individual microsporidian spindle pole bodies (mSPB) in each cell scales with meront nucleus volume. **(D)** The volume of mitosome groups associated with each individual mSPB scales with the mSPB volume. **(E)** The two mSPBs grow in concert. **(F)** Mitosome numbers increase gradually with the mSPB volume indicating ongoing mitosome scission (dotted line), whereas there appears to be a minimum mSPB size for the number of mitosomes at each mSPB, suggesting constraints on occupancy (continuous line; see text for discussion). **(G)** shows a maximum number of mitosomes on mSPB surface when modelled as a half sphere using stereological volume estimates. **(H)** Distribution of mitosomes across the two mSPBs. Even or near-even distributions are present in all but one cell (red numbers). N = 18 for (A, B, C, H); 36 for (D, F) and 7 for (G).

We next probed quantitative relationships between mitosomes and cell/organelle growth, looking for step changes during cell cycle regulation. Aggregate mitosome volume plotted against volume of cell, cytoplasm or nucleus (Fig 4A and Table 1) did not show abrupt changes (example in Fig 4A), whereas the correlations were strongest between mitosome volume and parasite nucleus volume. Interestingly, the correlation between mitosome volume and Golgi/ER (likely to be involved in cell wall and polar tube synthesis during spore development; not shown) was much weaker. The data are consistent with mitosome aggregate volume growing incrementally in concert with the cell and nucleus.

**Table 1.** Relationship between mitosome or mSPB volume/numbers and named compartments.

	r	*P*
Mitosome volume/number and cell compartments		
V mitosomes versus V cell	0.4593 (18)	0.0552
N mitosomes versus V cell	0.5258 (18)	0.0295
V mitosomes versus V nuc	0.6072 (18)	0.0075
N mitosomes versus V nuc	0.7642 (18)	0.0002
V mitosomes versus V cyto	0.6701 (18)	0.0023
N mitosomes versus V cyto	0.4905 (18)	0.0388
V mitosome (mean) versus V nuc	−0.1440 (18)	0.5686
mSPB volume and cell compartments		
V mSPB (mean) versus V cell	0.8083 (18)	<0.0001
V mSPB (mean) versus V nuc	0.8191 (18)	<0.0001
V mSPB versus V mitosomes	0.7297 (36)	<0.0001
V mSPB versus V mitosomes (mean)	0.2619 (18)	0.1228
V mSPB_1_ versus V mSPB_2_	0.7232 (18)	<0.001
V mSPB versus N mitosomes	0.5736 (36)	<0.001
S mSPB versus N mitosomes	0.9592 (7)	<0.001

N in parentheses. V, volume; N, number; nuc, nucleus; cyto, cytoplasm.

The number of mitosomes also scaled smoothly with cell and nuclear volume (Fig 4B), consistently indicating gradual mitosome multiplication. Accordingly, the average volume of individual mitosomes in cells remained relatively constant across cells with nuclei of different sizes (Table 1). The data indicate that the mitosomes in *E. cuniculi* grow and divide incrementally during meront interphase.

### Quantitative relationships between mitosome and mSPB

**Figure S5.**
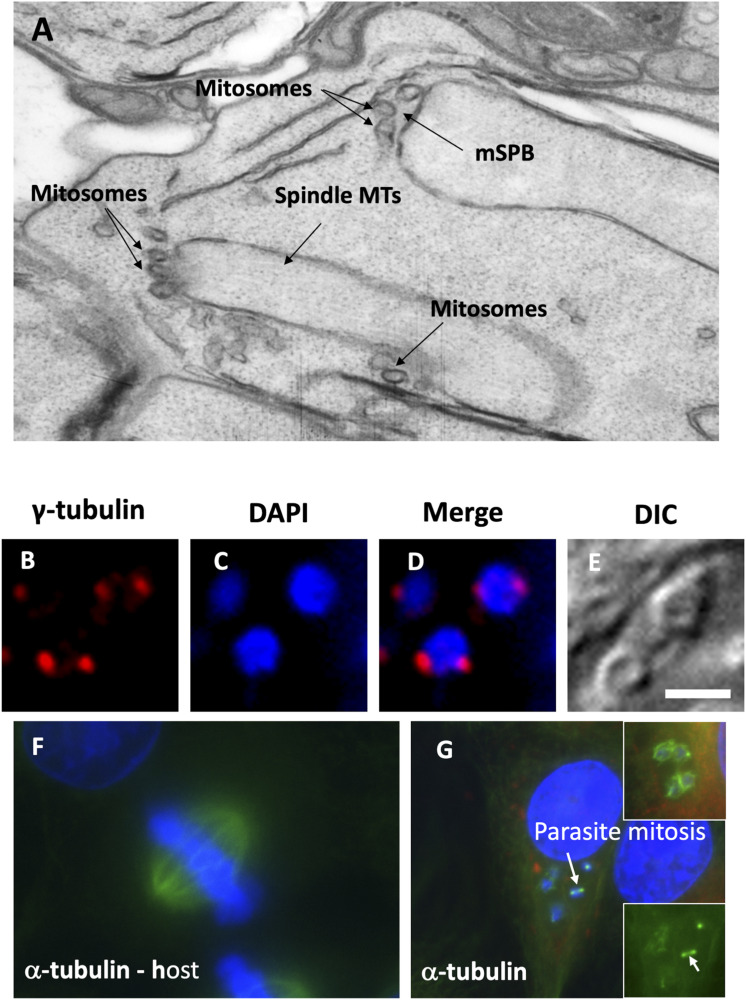
Mitosomes in mitosis and tubulin immunofluorescence. **(A)** Mitosomes are associated with microsporidian spindle pole bodies during in *E. cuniculi* mitosis (electron microscopy). Spindle microtubules (MTs) are visible inside the elongated nucleus with intact nuclear envelope in this dividing cell, which is a rare cell containing two nuclei. **(B, C, D, E)** Gamma-tubulin–positive structures (putative microsporidian spindle pole bodies) are present on either side of the *E. cuniculi* nucleus. **(F, G)** Alpha-tubulin labelling in open mitosis in RK13 and closed mitosis in *E. cuniculi*.

**Figure S6.**
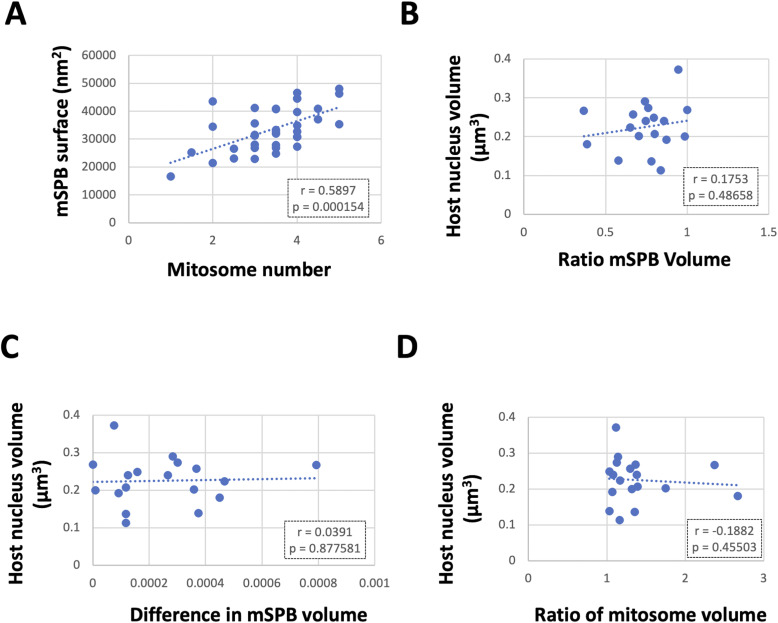
Mitosome size versus microsporidian spindle pole bodies (mSPB) surface and mitosome/mSPB homogeneity. **(A)** shows the relationship between mSPB surface (modelled) and mitosome number. **(B, C, D)** Explore the relationships between sizes of two mSPB or two mitosomal groups in each meront.

Most of *E. cuniculi* mitosomes were in direct contact with the amorphous mSPB structure in interphase (92%, N = 267). In rare mitotic meront profiles, all observed mitosomes were also located at the SPB (Fig S5). This association is consistent with a role for the mSPB in mitosome biogenesis and inheritance. To investigate this further, mSPB volumes (corrected for overprojection) were estimated in serial sections, and compared with the sizes of cellular compartments, mitosome or sister mSPBs (Figs 4 and S6, Table 1). The data showed mSPB size scaled with cell and nuclear volume and aggregate mitosome volume and mitosome numbers. However, the calculated mean mitosome volume at individual mSPBs did not correlate with mSPB size (Fig 4 and Table 1), and the distribution of mean mitosome volumes across all mSPBs was indistinguishable from a normal distribution (*P* = 0.073, N = 36, Shapiro–Wilk test).

These data suggested mitosome number (but not average volume) grows in concert the mSPB, but interestingly, there was a limit number of associated mitosomes for each size class of mSPB (continuous line in Fig 4F and extracted data in Fig 4G). For example, in terms of volume the data predicted an mSPB of 0.0002 µm^3^ would accommodate a single mitosome, whereas a 0.0006 µm^3^ mSPB can accommodate five mitosomes (modelled as a sphere); whereas in terms of surface, an mSPB area of 4,218 nm^2^ (Fig S6A) comfortably accommodates the “end” of a mitosome cylinder (diameter 58 nm; see above). Thus, the data indicate that, as mSPBs grow, they accommodate additional mitosomes that each grow out into the cytoplasm from a tethering zone.

### Even distribution of mitosomes across the two mSPBs

The strong association and quantitative relationship between mitosomes and mSPB structures in *E. cuniculi* suggested a role for mSPB in regulating of mitosome distribution in interphase and/or inheritance at cell division. To test this idea, we counted numbers of mitosomes across the two mSPBs present in each analysed nucleus. Strikingly, mitosomes were distributed as evenly as possible in 17 (Fig 4H) out of 18 meronts examined (statistically significant using weighted average analysis in a binomial distribution [Qiao et al, 2010]; highest *P* = 0.535 [limits 0.45–0.62]).

The even distribution of mitosome number could arise through tightly regulated partitioning at cell division/mSPB division or by unequal partitioning of mitosomes followed by compensatory multiplication. We therefore examined whether smaller cells (early in the cell cycle) had more asymmetry of the mSPBs or mitosomes but the data provided no evidence that smaller meronts (G1 cells) had more variation in mSPB sizes or mitosome number/volumes across the two mSPBs (Fig S6B–D). The data are therefore consistent with tight regulation of mitosome number through mSPB division followed by coordinated increase in mitosome number during the cell cycle.

### Phasic association of mitosomes with mSPB in *T. hominis*

Our data indicated mitosomes associate closely with the mSPB throughout the cell cycle in *E. cuniculi*, consistent with a role in mitosome biogenesis and inheritance. In *T. hominis*, development involves a phase of cell growth and nuclear multiplication (Ferguson & Lucocq, 2019) and mitosomes are more numerous. The resulting syncytium (also known as plasmodium) then cellularises to produce four, six or eight separate cells and multinucleate meronts can be classified as distinct morphotypes according to the thickness of the vacuole coat and whether they have cellularised.

**Figure 5.**
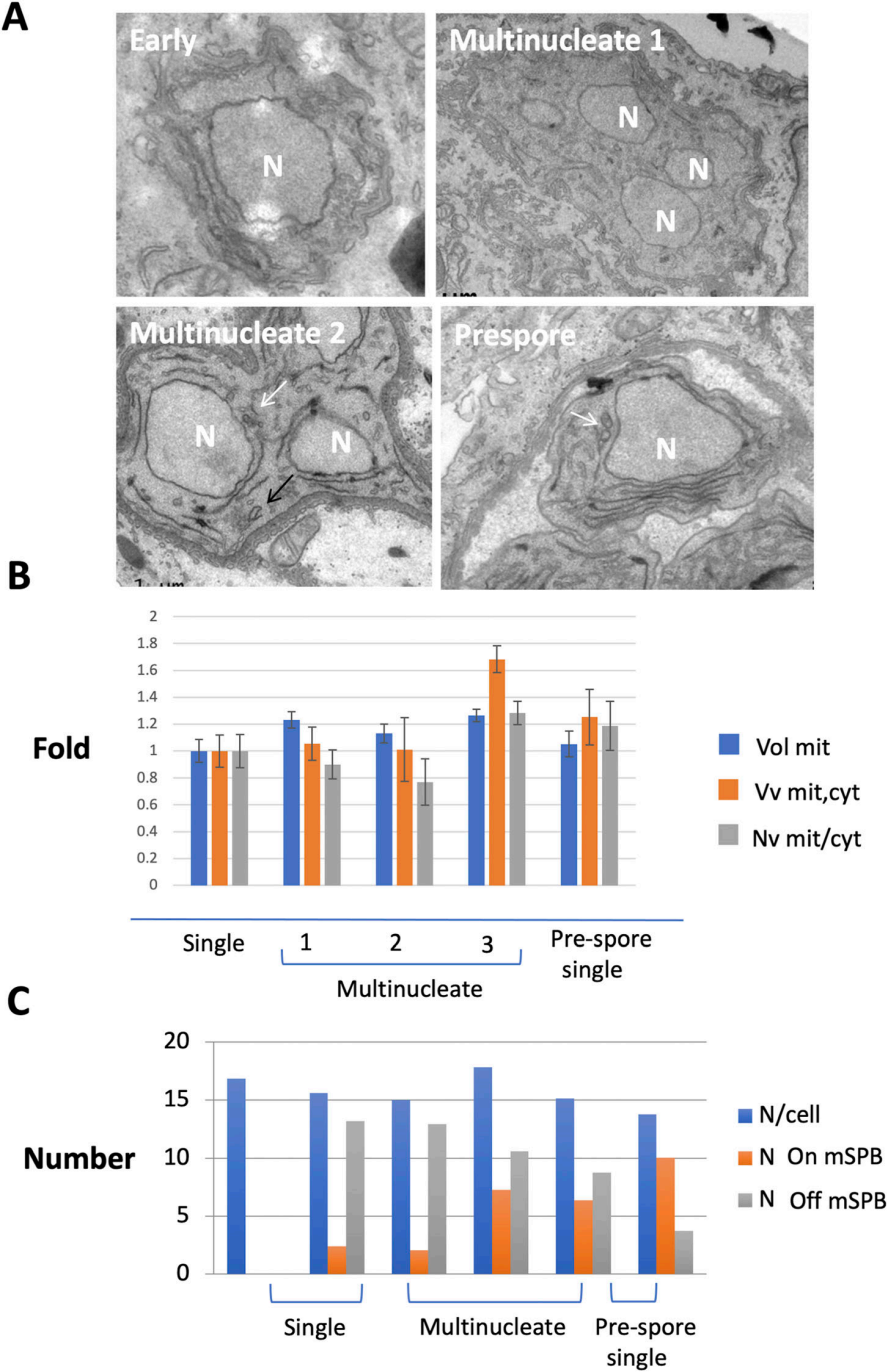
Dispersed mitosomes of multinucleate *T. hominis* meronts relocate to the microsporidian spindle pole bodies (mSPB) before cellularisation (electron microscopy). Qualitative and quantitative analyses of mitosomal localization during *T. hominis* development. **(A)** Ontogeny by conventional electron microscopy. Early meronts (Early) are found in the cytoplasm and undergo rounds of nuclear division to form multinucleate forms (multinucleate 1), around which a cytoplasmic coat then develops (multinucleate 2). As the coat thickens, a second membrane-like structure develops and forms a parisitophorous vacuole, within which cellularisation occurs forming individual sporonts (Prespore). N, nucleus; white arrows—juxtanuclear mitosomes and black arrows—peripheral mitosome. **(B, C)** Stereological analysis. Randomly positioned ministacks of 40 nm thick sections were used to estimate the number and size of mitosomes (mit) within the cytoplasm through stages of development. Absolute volume per nucleus (Vol mit), volume density (Vv mit, cyt), numerical density (Nv mit, cyt) and numbers of free mitosomes and of those located at the mSPB were estimated. Through the multinucleate stages, there is a progressive increase in the proportion of mitosomes associated with the mSPB. Multinucleate stages 1–3 were classified on the basis of coat thickness. For (A), error bars are SEM and N (the number of cells in ministacks used) for early, multinucleates 1, 2 and 3 and late: 11, 22, 8, 22, and 26, respectively.

Complete serial section analyses were not feasible for the larger *T. hominis* cells and so we used randomly placed ministacks of serial sections to quantify mitosomes and a stack of intermittent parallel sections to estimate cell and nucleus volume (see the Materials and Methods section and Williams et al [2002]; Ferguson et al [2017]). The data revealed *T. hominis* sampled from a mixed infection population contains a rather constant number of mitosomes (∼16 per cell), with numbers varying between 14 and 18/cell; Fig 5). In contrast to *E. cuniculi*, the proportion of mitosomes associated with the mSPB varied according to the cellular stage of development (Fig 5C). In single cells, early in the developmental programme, most mitosomes were distant from the mSPB, but the association increased progressively through the syncytial stages. After cellularization, 73% of the mitosomes were now associated with the mSPB. Thus, mitosomes undergo progressive association with the mSPB as meronts with multiple nuclei approached cellularisation.

**Figure 6.**
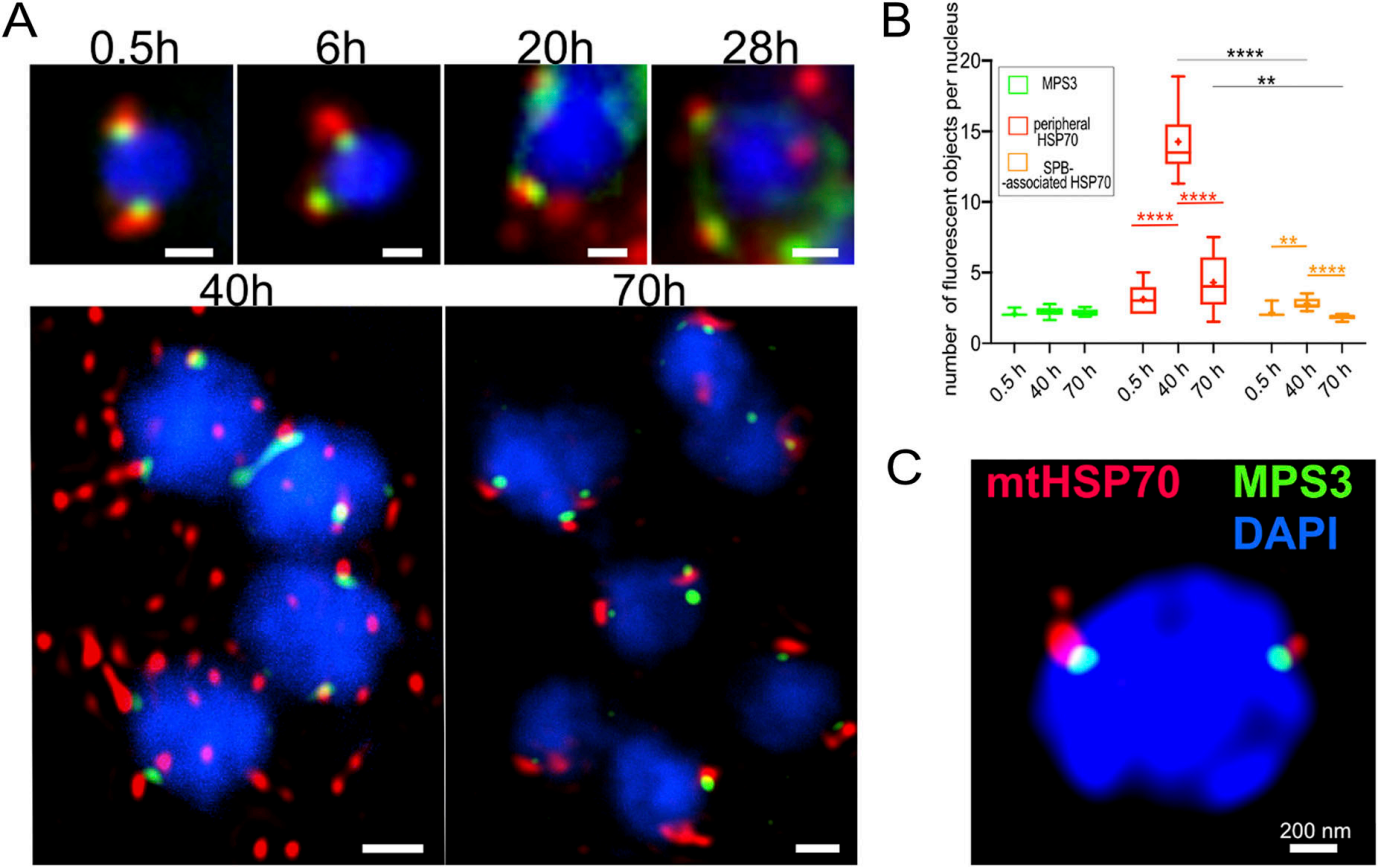
Dispersed mitosomes in *T. hominis* meronts redistribute during development (immunofluorescence). **(A)** Overview of mitosome and SPB distribution during *T hominis* development. Purified spores were seeded onto uninfected RK13 (0 h) to initiate synchronized time course of infection and samples were taken at 0.5, 6, 20, 28, 40, and 70 h post infection before fixation and immunofluorescence microscopy and stained using antibodies for the mitosome marker *T hominis* HSP70 (ThmtHSP70), microsporidian spindle pole bodies marker MPS3 (ThMPS3). Images are maximum intensity Z-projections of widefield fluorescence Z-stacks (0.5–28 h, and Fig S7), or of deconvolved confocal Z-stacks (40 and 70 h). Scale bars correspond to 500 nm. **(B)** Quantification of the detected fluorescent volumetric objects (Fig S8) corresponding to MPS3-SPB (green); MPS3-associated mitosomes (red), and peripheral mitosomes (orange). *T* test was used to test the significance of the difference between two sample means, displayed above the plots (****P ≤ 0.0001, ***P ≤ 0.001, **P ≤ 0.01, *P ≤ 0.05, not significant *P* > 0.05). **(C)** Deconvolved STED super-resolution image of the *T. hominis* nucleus inside the parasite cell labelled with antibodies against mtHSP70 (red) and MPS3 (green).

**Figure S7.**
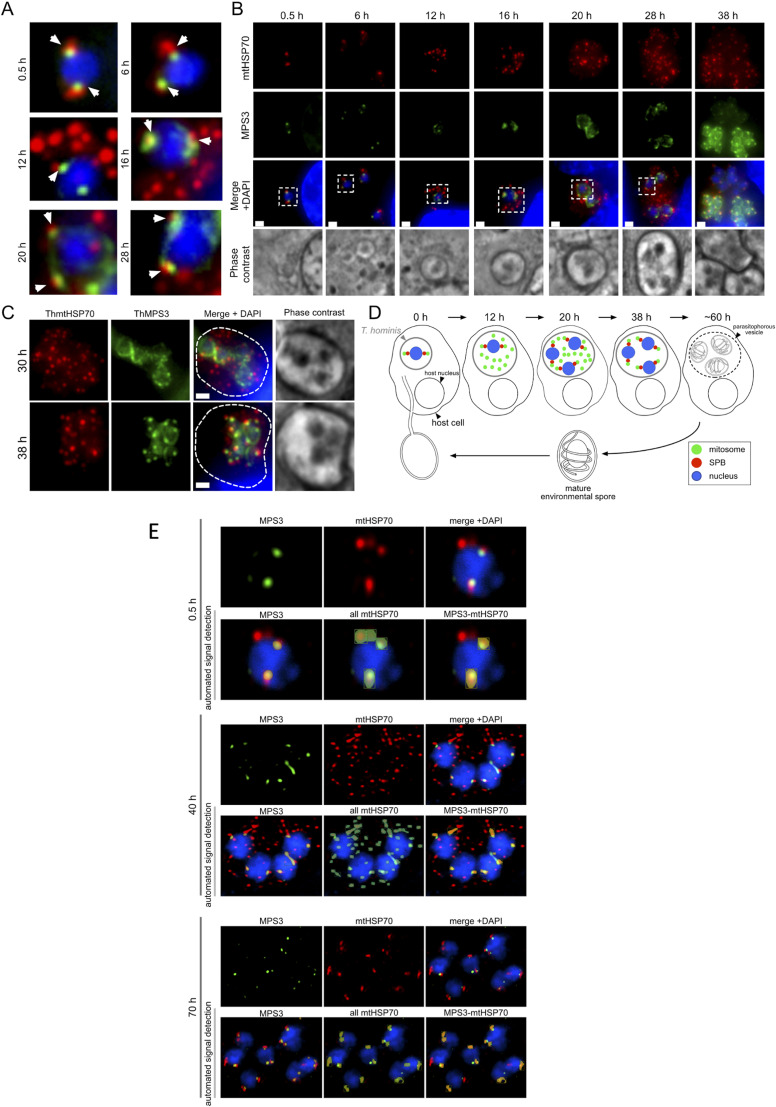
Distribution of mitosomes and SPBs observed inside *T. hominis* cells across the synchronised time course of infection. **(A, B, C)** Images of the *T. hominis* cells (blue) labelled with the antibodies against *Th*mtHSP70 (red) and *Th*MPS3 (green) observed in the different time points of the synchronised time course of the infection. Subpopulations of the MSP3-associated mitosomal HSP70 fluorescence (white arrowheads) were observed in all time points. **(A, B)** The images presented in (A) are cropped versions of the images presented in (B), the cropped areas were annotated (white dashed rectangles). **(C)** 38 h post infection, first cells displaying an apparent change in distribution of the mitosomes (bottom panel) were observed. **(D)** A model representing distribution patterns of the mitosomes (green) and the SPBs (red) inside *T. hominis* cells (grey) at different time points of RK-13 host cell (black) infection. All images were acquired using widefield fluorescence microscope. Cell nuclei were labelled with DAPI. Scale bars correspond to 1 μm. **(E)** Automated detection of fluorescent volumetric objects. Z-stacks of laser scanning confocal images of *T. hominis* cells from 0.5, 40, and 70 h timepoints were deconvolved and analysed in VoloCity software package (Perkin Elmer). The first row of each panel is a maximum-intensity Z-projection of the deconvolved Z-stack. The second row of each panel represents the automated signal detection results from VoloCity. The fluorescent volumetric objects were automatically detected using “find objects” function (minimum object size 0.01 μm^3^) in a red fluorescence channel (all mtHSP70 highlighted in green) or green fluorescence channel (MPS3 highlighted in yellow). MPS3 and mtHSP70 objects that were at least partially overlapping (MPS3-mtHSP70 highlighted in orange) were detected using “exclude non-touching” function implemented in VoloCity. Scale bars correspond to 1 μm.

These EM studies were done on non-synchronised cells and we attempted to synchronise infections by applying purified spores to uninfected RK13. In this case, the infected parasites were sparse and so we used immunofluorescence localisation with markers for mitosomes (HSP70) and mSPB (MPS3; Figs 6 and S7). IF cannot distinguish individual mitosomes when clustered at the mSPB; see for example Fig S1A–C; but the data do report on mitosome distribution. Broadly, the data confirm that early dispersal of mitosomes (at 40 h; Figs 6 and S7) occurs before progressive association with the mSPB during vacuole formation and cellularisation (70 h). During the dispersal phase, the number of peripheral mitosome signals corresponded to those we detected using quantitative EM but reduced as they clustered at the mSPB. Immediately post infection (a stage not found in our EM sample), mitosomes were also located predominantly at the mSPB resembling the distribution in *E. cuniculi*.

### Microtubules and mSPB in mitosome positioning

Centrosomes are a major focus of microtubule nucleation in animal and yeast cells. In *E. cuniculi*, MTs were only occasionally observed fanning out from the mSPB into the cytoplasm of interphase cells (not shown) but MTs were numerous within the nucleoplasm of mitotic profiles, consistent with intra-nuclear mitosis (Fig S5; Boettcher & Barral, 2013; Keeling & Fast, 2002). In IF, antibodies to highly conserved α-tubulin produced signals on either side of the nucleus of mitotic profiles and also as cytoplasmic signals in interphase (Fig S5F and G).

**Figure S8.**
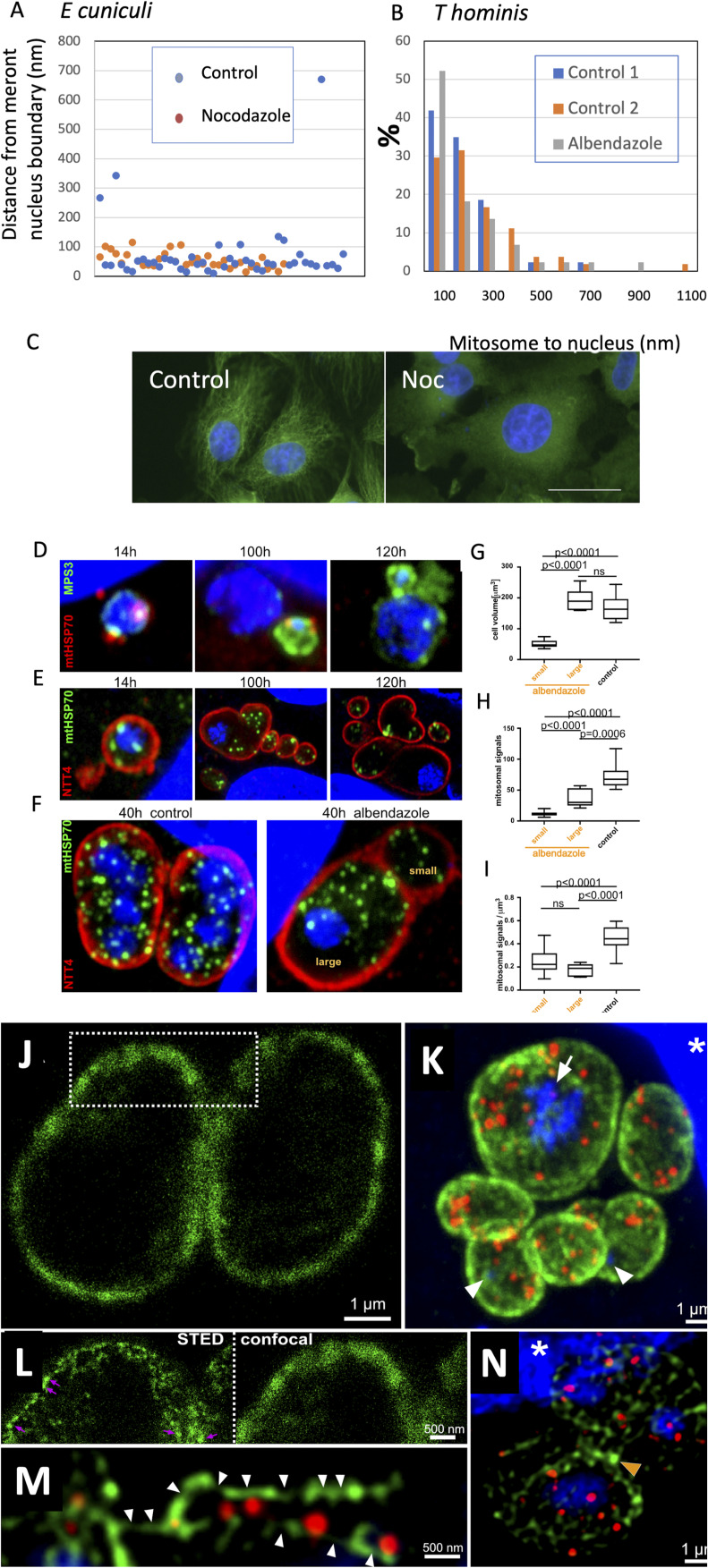
Mitosome positioning after treatment with antimicrotubule drugs. **(A, B)** Electron microscopy data. Infected monolayers of RK13 cells were treated with 10 µg nocodazole ((A), *E*. *cuniculi*) or 10 ng/ml of Albendazole ((B), *T*. *hominis*; see the Materials and Methods section) and processed for conventional epoxy resin microscopy. Mitosomes were identified as double-membraned structures with homogeneous matrix and distance to the nearest nuclear envelope measured. In *E. cuniculi* peripheral mitosomes were no longer detected after nocodazole. In (A), N = 46 for control and 32 for Noc. In (B) N = 43 and 54 for controls 1 and 2, respectively, and 44 for Noc. **(B)** In (A) Chi square 0.16 df 1 *P* > 0.2 and in (B) Control 1 versus Albendazole Chi square 5.4 df 3 *P* > 0.1 and Control 2 versus Albendazole Chi square 7.0 df 3 *P* > 0.1. **(C)** effect of nocodazole on microtubule network in RK13. Cells were treated with DMSO carrier alone (Control) or 10 µg nocodazole (Noc) for 2 h and immunolabeled for tubulin as described in the Materials and Methods section. **(D, E, F, G, H, I)** Light microscopy data. **(D, E)** *T. hominis*-infecting RK-13 host cells were treated with albendazole throughout the synchronised time course of the infection. **(D, E)** Methanol–acetone fixed samples were collected across the time course and labelled with specific antibodies: rabbit anti-mtHSP70 ((D), red) and rat anti-MPS3 ((D), green); or rat anti-mtHSP70 ((E), green) and rabbit anti-NTT4 ((E), red). **(F)** Representative images of the parasite cells observed after the first cellular division in the albendazole treated sample (right panel) and the non-treated control (left panel). At 40 h post infection time point, all parasite cells in the infected host cell monolayer have undergone the first cell division. Pairs of the parasite cells were analysed only if two parasite cells were observed inside a single host cell. The (large) *T. hominis* cell with the nucleus, and the (small) anucleate parasite cells observed after the first asymmetric cytokinesis in the albendazole-treated samples were indicated. **(G, H, I)** Quantitative data. **(F, G, H, I)** Volume of the *T. hominis* cells (G) observed in the albendazole treated (small and large) and the non-drug-treated (control) samples was measured based on the cell outline detected with antibodies against the membrane protein ATP transporter NTT4 ((F), red) in confocal Z-stack of the parasite cells. Number of the mtHSP70 objects per cell (H) corresponds to the number of the detected mtHSP70 fluorescence volumetric objects ((F), green) inside *T. hominis* cells. Numbers of mtHSP70 objects per cell volume (I) were quantified for each individual cell by dividing the number of the mtHSP70 fluorescent objects inside a parasite cell by the measured volume of the cell. *T* test was used to test the significance of the difference between two sample means, displayed above the plots (*P* ≤ 0.0001, *P* ≤ 0.001, *P* ≤ 0.01, *P* ≤ 0.05, ns, not significant, *P* > 0.05). **(J, K, L, M)** Actin cytoskeleton of *T. hominis* is not affected by the albendazole treatment. **(J)** A single confocal section through two late *T. hominis* meronts inside RK13 host cell labelled with the specific anti-*T. hominis* actin (green) antibodies. **(K)** Maximum intensity projection of a Z-stack of confocal images through *T. hominis* infected RK13 cell from an albendazole treated sample labelled with anti-Th-actin (green) and anti-ThmtHSP70 (red). Large nucleus inside a “mother cell” (arrow) and a DAPI-stained pseudo-nuclei (arrowheads) inside the small anucleate “daughter cells” were indicated. **(J, L)** STED super-resolution images of the *T. hominis* cortical actin cytoskeleton ((J), dashed square). Actin-enriched spots reminiscent of “actin patches” were indicated (arrows). **(M)** A single deconvolved confocal section through structures reminiscent of the actin filaments (arrowheads) often observed in close proximity to the mtHSP70 mitosomal signals (red) inside the *T. hominis cells*. **(N)** Actin division ring-like structure at the septum of the dividing *T. hominis* meront (orange arrowhead). White asterisks indicate RK13 host cell nuclei.

To analyse dependence of mitosome mSPB association on microtubule integrity, *E. cuniculi*-infected RK13 cells were incubated with nocodazole (10 μg/ml for 4 h), a treatment which induced homogenization of α-tubulin staining in RK13 by IF (Fig S8) consistent with microtubule depolymerization. In electron microscopy, this treatment did not induce detectable dislocation of mitosomes from the mSPB (Fig S8A). Similarly, in *T. hominis*, meronts treated with the microtubule depolymerizing agent albendazole (10 ng/ml, 3 h) did not increase the mitosome to mSPB distance using EM (Fig S8B).

We also investigated the effects of albendazole in *T. hominis* cells using immunofluorescence light microscopy after spore infection (Fig S8) and extended drug treatments (14–100 h; see below). DAPI-stained nuclei of the albendazole-treated parasite cells increased in diameter having failed to undergo fission, consistent with the expected role of microtubules in the nuclear division during the closed mitosis in microsporidia. Specifically, at 100 h post infection, when control cells had their mitosomes predominantly located at the mSPB, small portions of DAPI-positive chromatin appeared to “bud-off” from a large nucleus. These newly formed “pseudo-nuclei” often had mitosomes attached to a MPS3-positive mSPB indicating the mitosome–SPB association was stable and not mediated via microtubules which were depolymerised by the albendazole. Interestingly, at 40 h (when control cells had multiple nuclei surround by largely peripheral mitosomes), albendazole treatment produced enlarged cells with a single enlarged nucleus surrounded by peripheral mitosomes, suggesting that microtubules do not have an active role in maintaining a peripheral mitosome population. On the other hand, the large 40-h cells appeared to have generated nuclei-free “mini-cells” which consistently contained widely distributed mitosomes, reflecting a stochastic distribution into these newly formed cytoplasmic units. Cortical actin cytoskeleton was maintained in the albendazole-treated *T. hominis* cells and did not colocalise with all peripheral mitosomes, which is again consistent with stochastic segregation (Fig S8J–N).

In summary, the microtubule inhibitor data indicated that microtubule-based movement/positioning is unlikely to explain the association of mitosomes with the mSPB in both the microsporidian species investigated here.

### Identification and sequence analysis of microsporidian dynamin-related proteins

BlastP and tBlastN (*E*-value thresholds = 0.05 with *S. cerevisiae*, *T. hominis*, *E. cuniculi*, and *Rozella allomycis* dynamin-related protein (DRP) sequences as queries), and HMMER (−10 × 10^−05^, hmm profile queries: Dynamin_M PF01031.23, Dynamin_N PF00350.26, GTPase effector domain [GED] PF02212.21) searches were used to identify microsporidian homologues of dynamin in available microsporidian genomes.

**Figure 7.**
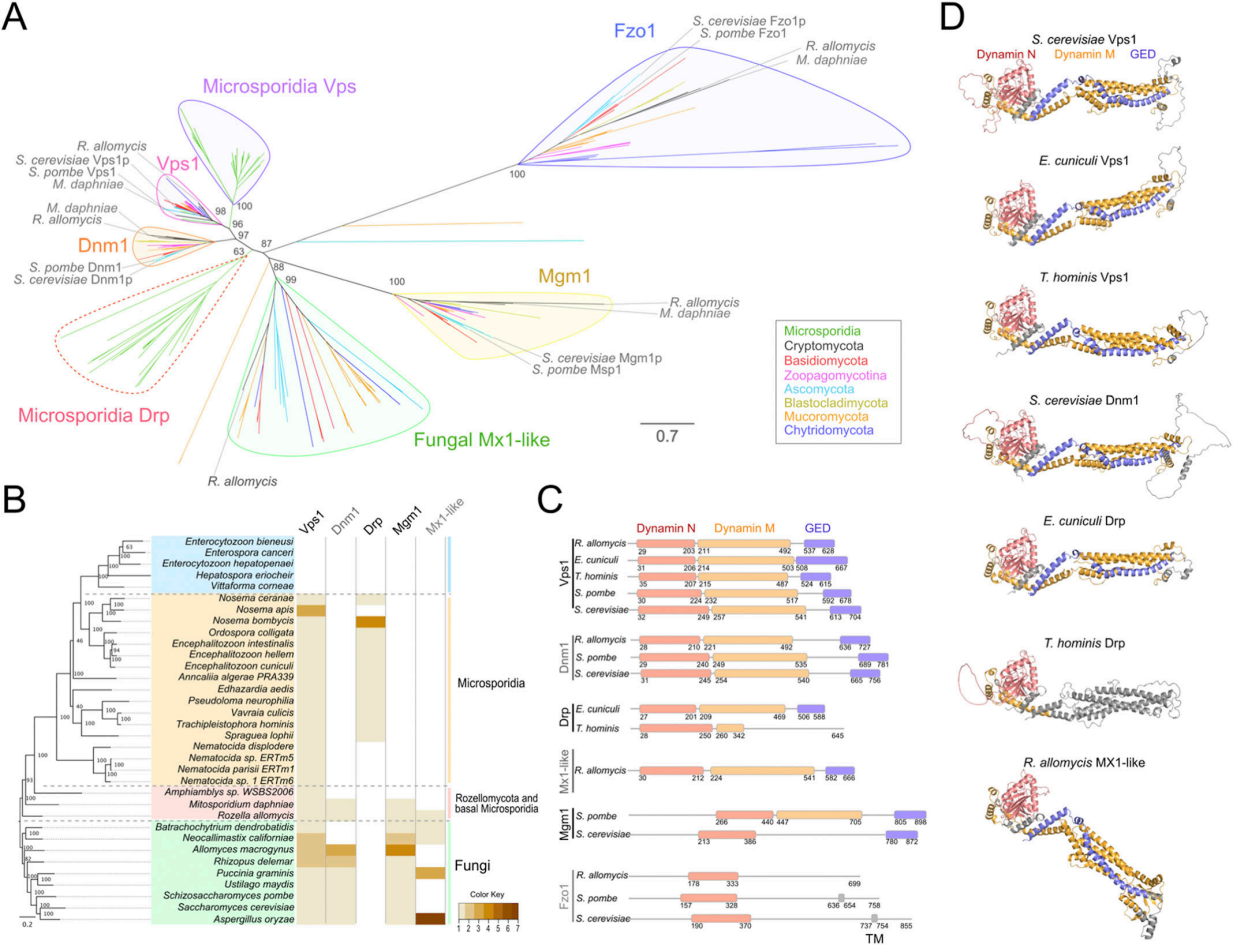
Conservation of microsporidian dynamin-related proteins. **(A)** Maximum likelihood tree of dynamin homologues identified in microsporidia and fungi using BlastP and HMM profile searches. More conserved microsporidian dynamin related protein (DRP) sequences (microsporidian VPS) formed shorter branches and grouped together with their fungal homologues including *S. cerevisiae* Vps1p and *S. pombe* Vps1 with high support (96%). The more divergent (longer branches; and lower sequence conservation, Fig S11) group of microsporidian DRPs (microsporidian Drp, Red dashed line) formed a distinct group with a weakly supported position (63%) at the base of the tree between strongly supported Dnm1/Vps1 group and those of Mgm1 and fungal dynamins resembling human Mx1 (Mx1-like). Sequences of Vps1, Dnm1, Mgm1, and Fzo1 from two species closely related to microsporidia (*R. allomycis* and *M*. *daphniae*) grouped together with their fungal orthologues. Distantly related Fzo1 homologues were used as an outgroup. Branches of the tree were coloured to indicate dynamins from major fungal lineages (legend). Rapid bootstrap value support values (% of 1,000 replicates) were displayed only for the key branches. Scale bar represents number of substitutions per site. **(B)** Distribution of the identified DRPs was mapped onto a species tree of microsporidia (orange and blue), their closest relatives including *R. allomycis* (red), and a selection of Fungi (green). Heatmap represent the number of homologues (Colour key) identified in each genome. Most of microsporidia with no DRP homologues identified in our analysis (blue) belong to lineage of microsporidia which have lost the glycolytic pathway. **(C)** Domain architecture of dynamins used in the phylogenetic analysis. Microsporidian DRPs including *E. cuniculi* and *T. hominis* Vps1, and *E. cuniculi* Drp have conserved domain architecture containing GTPase (Dynamin N), middle (Dynamin M) and GTPase effector domains (GED). The region between Dynamin, M and GED domains corresponding to pleckstrin homology domain (human dynamin1, plant Drp3A) and insert B (in human Drp1) appears to be considerably reduced in microsporidia and is shorter than that in *S. cerevisiae* Vps1p. **(A)** In *T. hominis* Drp only a short Dynamin M fragment and no GED domains were identified in Interproscan analysis further supporting high level of divergence observe in microsporidian DRPs ((A), and Figs S9 and S10). **(D)** AlphaFold2 structure predictions demonstrating conservation of a general dynamin-like fold architecture in all identified microsporidian DRPs.

**Figure S9.**
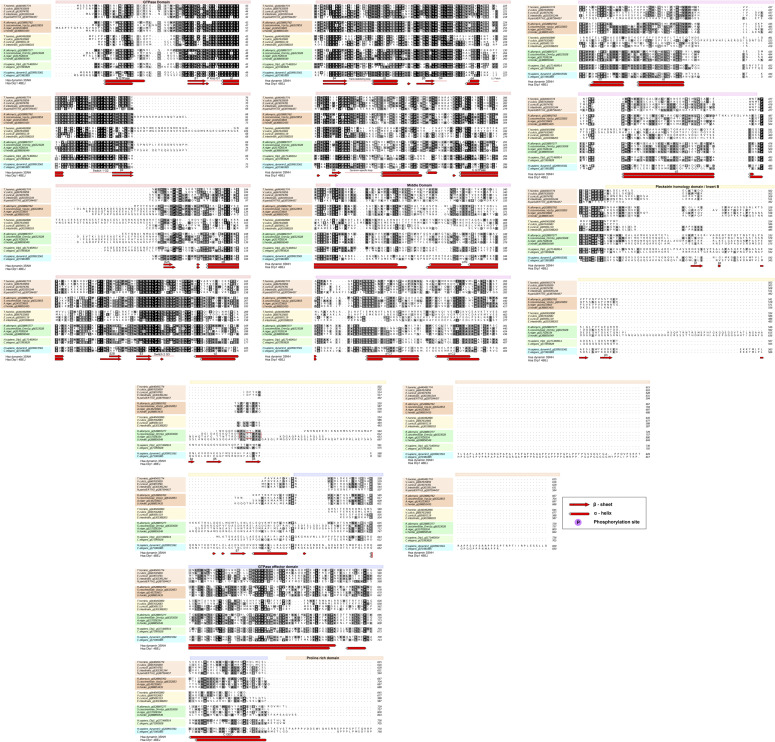
Multiple sequence alignment of representative dynamin and dynamin-related protein homologue sequences from fungi, microsporidia, and Metazoa. Coloured boxes above alignment annotate domain architecture according to available crystal structures. Secondary structures below the alignment correspond to human dynamin1 (pdb 3SHN), human DNM1L (pdb 4BEJ), and secondary structure predictions for *E. cuniculi* Vps1p orthologue generated using Jpred 3 server (Cole et al, 2008) (www.compbio.dundee.ac.uk/jpred/). Alpha-helices are presented as cylinders and beta strands are presented as arrows. Red box indicates sequence motif responsible for interaction of Dnm1p with Mdv1p adaptor protein in *S. cerevisiae* (Bui et al, 2012). Groups of sequences were annotated with colours corresponding to those used in the trees. Alignment was formatted in ALINE (Bond et al, 2009), conserved residues were coloured using “colouring by similarity” function implemented in ALINE with 0.1 low similarity cut-off. Sequences used in the alignment: *Trachipleistophora hominis* Drp1 (GI 440492890), *T. hominis* Vps1p (GI 440491774), *Vavraia culicis* Drp1 (GI 667632683), *V. culicis* Vps1p (GI 667635859), *E, cuniculi* Drp1 (GI 85691119), *E. cuniculi* Vps1p (GI 19074781), *Encephalitozoon intestinalis* Drp1 (GI 303388203), *E. intestinalis* Vps1p (GI 303391244), *N. parisii* ERTm3 Vps1p (GI 387594457), *Saccharomyces cerevisiae* Vps1p (GI 6322853), *S. cerevisiae* Dnm1p (GI 6323028), *Rozella allomycis* Vps1p (GI 528892762), *R. allomycis* Dnm1p (GI 528897277), *Aspergillus niger* Vps1p (GI 145233603), *A. niger* Dnm1p (GI 317028334), *Ustilago hordei* Dnm1p (GI 388856549), *U. hordei* Vps1p (GI 388853435), *Homo sapiens* Dlp1 (GI 171460914), *H. sapiens* dynamin 3 (GI 209915561), *Caenorhabditis elegans* dynamin (GI 71981885), *C. elegans* Dlp1 (GI 71993828).

**Figure S10.**
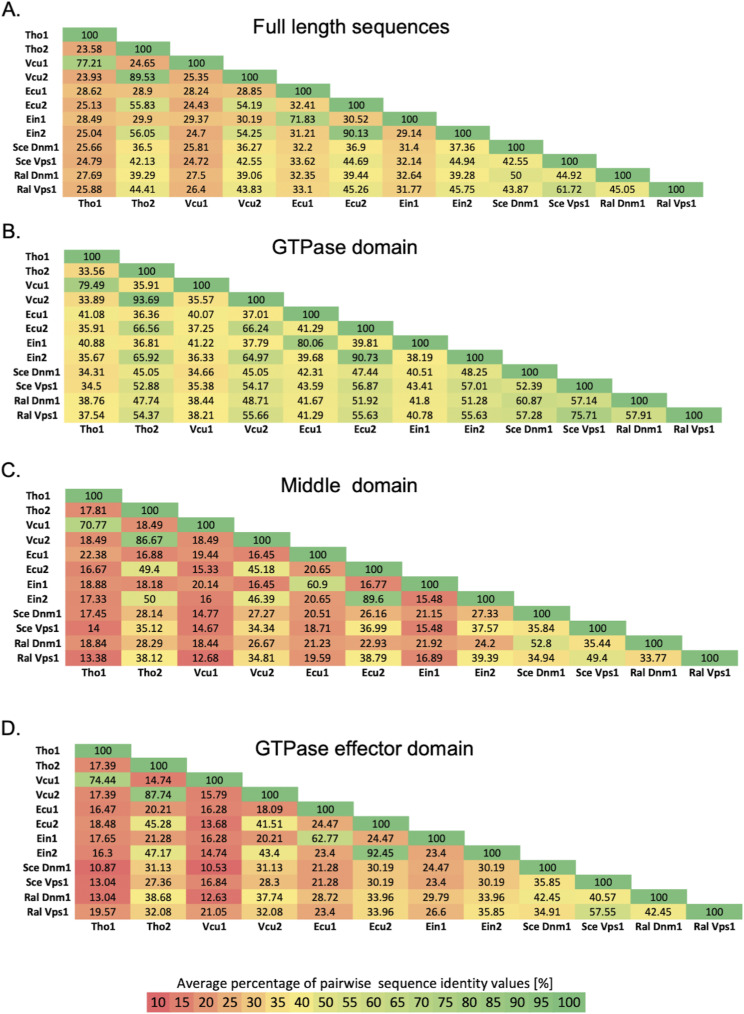
Pairwise percentage of sequence identity. **(A, B, C, D)** Pairwise percentage of sequence identity for full-length sequences (A), GTPase domain (B), middle domain (MD) (C), and GTP-ase effector domain; (D) within the alignment were calculated using -output = sim function implemented in T-coffee.

Comparison of a multiple sequence alignment of microsporidian DRPs with fungal (Dnm1p and Vps1p) and with metazoan (Drp1 and dynamin3) homologues (Figs 7 and S9) indicates that microsporidian homologues seem to have three of the conserved domains: GTPase domain, middle domain (MD), and GED. The sequence region corresponding to the Pleckstrin homology domain in classical dynamins or Insert B in mammalian Drp1 and yeast Dnm1p did not align well and appears to be either significantly reduced or lost in all of the microsporidian putative DRPs (Figs 7 and S9). The microsporidian GTPase domain is the best conserved domain within the multiple sequence alignment of DRPs from four microsporidian species (*T. hominis*, *E. cuniculi*, *Encephalitozoon intestinalis*, and *Vavraia culicis*), and shares considerably higher pairwise percentage of sequence identity with *S. cerevisiae* and *R. allomycis* than the MD and GED domains (Fig S10). Alphafold2 structure predictions for the two DRPs from *E. cuniculi*, *T. hominis*, and *S. cerevisiae* (Fig 7D) suggest that despite low-sequence identity, the overall fold architecture including α-helices and β-strands of GTPase, MD and GED are conserved between them and dynamins that have solved crystal structures including human dynamin1 (pdb 3SNH) and human Drp1 (pdb 4BEJ). Sequence regions corresponding to Dynamin M and GED seems to be the most highly divergent in *T. hominis* Drp as InterProScan searches failed to detect the conserved sequence motifs present in other DRPs (Fig 7C).

Analysis of average pairwise percentage of sequence identities between full-length proteins within the alignment revealed that one of the DRPs from each microsporidian species shared 24.7–33.6% identity with sequences from *S. cerevisiae* and *R. allomycis*, whereas the second microsporidian homologue shared 36.3–45.8% identity with them (Fig S10).

In phylogenetic analysis, the more conserved microsporidian DRP groups strongly with fungal Vps1 sequences (Fig 7A). The second, less conserved (Figs 7A and S10) and less widely distributed (Fig 7B) microsporidian DRP has weakly supported (63% rapid bootstrap value) position at the base of the DRP phylogenetic tree (Fig 7A), therefore its origin remains unclear. Based on the closer similarity of its domain architecture to that of Dnm1, Vps1, and MX1-like, and loss of the Mx1-like coding gene in *Mitosporidium daphniae*, the most parsimonious origin of the second microsporidian DRP would be its divergence from Dnm1. However, an origin from Mx1-like, Mgm1 or duplicated copy of Vps1 cannot be excluded. The presence of all major DRP homologues (Dnm1, Vps1, Mgm1) in *R. allomycis*, *M. daphniae*, and most of the analysed fungi is consistent with the presence of these DRPs in the last common ancestor of all fungi. This ancestral DRP repertoire seems to have been subsequently degraded during microsporidian reductive evolution (Fig 7B). Strikingly, in the most divergent microsporidian lineage, including species with degraded glycolytic pathway (Wiredu Boakye et al, 2017), all DRPs seem to have been lost (Fig 7B).

### Detection of microsporidian homologues of yeast and human Dnm1p/Drp1 interaction partners

**Figure S11.**
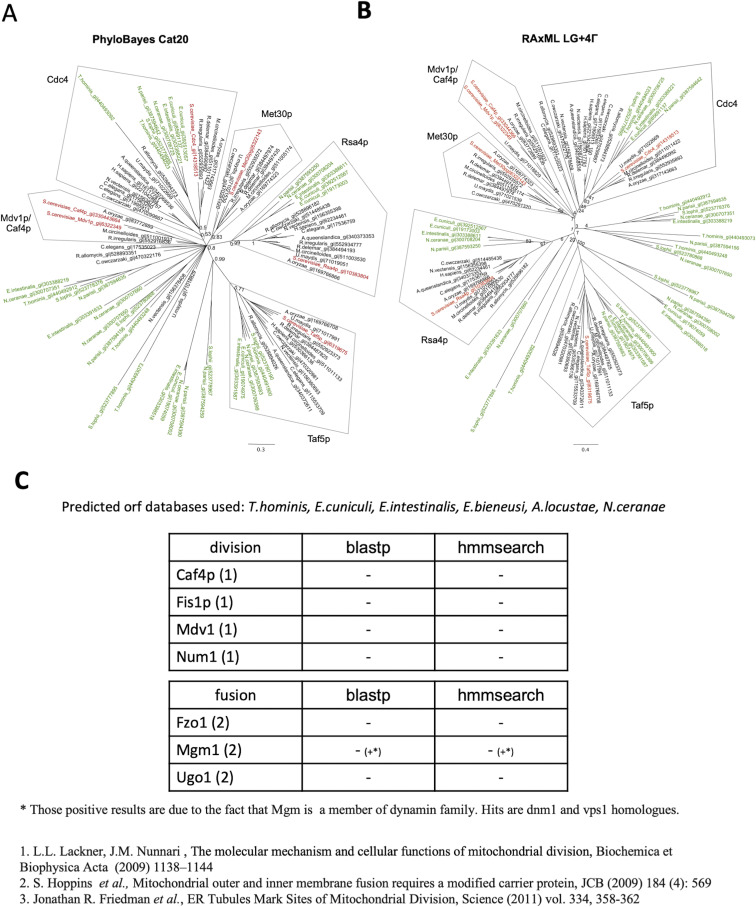
Phylogenetic analysis of WD40 domain sequences from microsporidia, fungi, and Metazoa. **(A, B)** Bayesian tree was generated with PhyloBayes (Lartillot et al, 2009) under C20 (Quang et al, 2008) model and (B) maximum likelihood tree was generated using RAxML (Stamatakis et al, 2005) under LG+4Γ. **(A, B)** Support values for key nodes of the trees correspond to (A) Bayesian posterior probabilities and (B) bootstrap values from 100 rapid bootstrap replicates. Scale bar represents the number of substitutions per site. Sequences used in the analysis were selected as follows. Database of *S. cerevisiae* protein sequences (NCBI) was searched using BlastP (Altschul et al, 1990) with *S. cerevisiae* Mdv1p and Caf4p as queries and six best BlastP hits including queries were selected for the analysis. Subsequently, BlastP searches with the six *S. cerevisiae* WD40 proteins as queries were performed against other fungi and Metazoa and the best hits were selected. The first six best BlastP hits were selected from Mdv1p and Caf4p searches against microsporidia. The selected sequences were aligned with MUSCLE (Edgar, 2004) and trimmed using trimAL (Capella-Gutiérrez et al, 2009) with gappyout method. Trimmed alignment had 230 positions and corresponded only to the WD40 region of the proteins. **(C)** Homologues of mitochondrial division and fusion proteins (dnm not included) in microsporidian genomes.

We investigated the presence of genes encoding other components of the mitochondrial scission machinery in *T*. *hominis* and *E*. *cuniculi* genomes. BlastP (Altschul et al, 1990; Fig S11) and more sensitive HMM (hidden Markov models) profile searches (Eddy, 1998) were used to search for potential microsporidian homologues of proteins proposed to be involved in recruitment of Dnm1p or Drp1 to mitochondria of yeast (Caf4p, [Griffin et al, 2005]; Mdv1p, [Tieu et al, 2002]; Fis1p, [Mozdy et al, 2000]; Num1p, [Cerveny et al, 2007; Lackner et al, 2013]; Mdm36p, [Hammermeister et al, 2010; Lackner et al, 2013]) or mammals (MFF, [Otera et al, 2010]; Mid49, MiD51 [Palmer et al, 2011] and Fis1, [Stirnimann et al, 2010]). Both methods only detected microsporidian proteins containing WD40 domains (Stirnimann et al, 2010) that are also found in two yeast homologues Mdv1p and Caf4p.

Caf4p and Mdv1 were previously shown to cluster together with microsporidian sequences in published analysis of clusters of homologues from 11 microsporidian, 7 fungal, and 3 animal genomes (Nakjang et al, 2013). Examination of the cluster (c_1599) containing Caf4p and Mdv1 revealed that it consists of 1,054 protein sequences containing WD40 repeats (InterProScan 5) (Jones et al, 2014) including 229 microsporidian proteins. Examination of the pairwise alignments generated by BlastP revealed that proteins align only in the C-terminal region of Caf4p and Mdv1p containing the WD40 repeats. No reciprocal best BlastP hits were identified between microsporidia and *S. cerevisiae* when Mdv1p and Caf4p were used as initial queries (Fig S11).

In maximum likelihood and Bayesian phylogenetic trees, Caf4p and Mdv1p grouped together with orthologues from other fungi (Fig S11). None of the microsporidian proteins used in construction of the WD40 domain phylogenies grouped together with Mdv1p and Caf4p. HMM profile searches have also detected microsporidian proteins containing tetratricopeptide repeat motifs; however, none of the tested hits (*E*-value < 0.001) contained a predicted C-terminal trans-membrane domain like that present in yeast Fis1p and human Fis1 (Mozdy et al, 2000; Tieu et al, 2002).

### Dynamin inhibitors restrict mitosome fission and microsporidian dynamin homologues rescue mitochondrial constriction function in dnm1-deficient yeast

**Figure S12.**
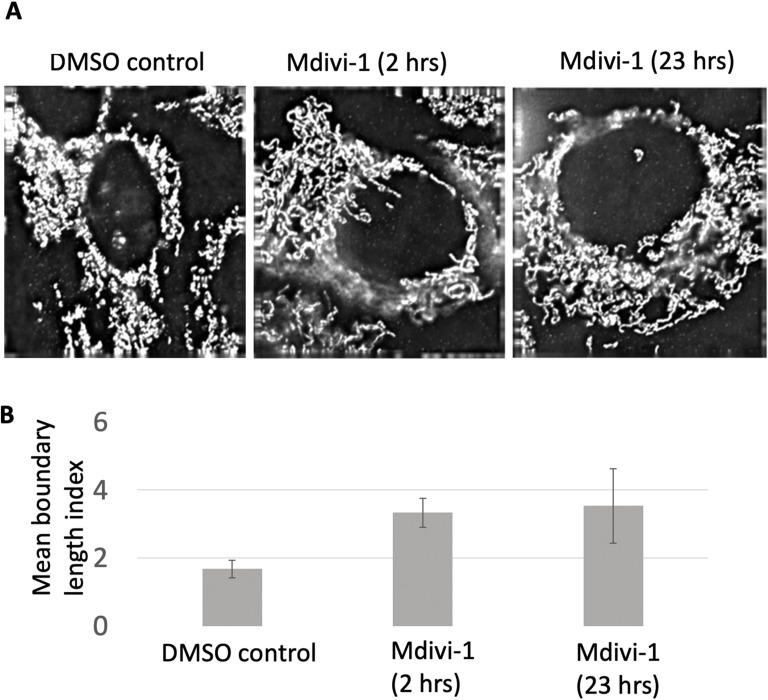
Mdivi1 inhibits mitochondria scission in RK13 cells. Immunolocalisation of mitochondria in uninfected RK13 with F0-F1 ATP-ase monoclonal antibody. **(A)** Left—DMSO treated control cells (1:1,000 dilution). Middle and right—Mdivi-1 (50 μM)-treated mammalian cells and incubated for 2 and 23 h, respectively. **(B)** Stereological data quantification. Tukey’s multiple comparison test was used in one-way ANOVA. Mdivi-1 (2 and 23 h) >90% increase in boundary length as determined by ImageJ. Significant difference was observed between control (0.045 ± 0.0069 SEM) and Mdivi-1 (*P* < 0.05) with no difference between the Mdivi-1 treated cells (*P* > 0.05). Columns represent an average of 15 randomly acquired cells. Error bars represent SEM.

To test the roles of mitochondrial dynamins in mitosome biogenesis, we treated RK13 cells infected with *E*. *cuniculi* or *T*. *hominis* with Dynasore (Macia et al, 2006) and Mdivi-1 (Cassidy-Stone et al, 2008; Smith & Gallo, 2017). Mdivi-1 interferes with oligomeric assembly by binding outside the GTP-ase domain, thereby inhibiting GTP-ase activity (Cassidy-Stone et al, 2008; Smith & Gallo, 2017) in vitro without inhibiting mitochondrial fusion. Mdivi-1 targets both mammalian and yeast dnm-1 (Cassidy-Stone et al, 2008). Dynasore is a small molecule, selective noncompetitive inhibitor of the protein dynamin 1, dynamin 2, and Drp1 and may also display activity against VPS-like dynamin (Preta et al, 2015). Dynasore inhibits the GTPase activity of dynamin, blocking constrictions and fission. To confirm that Mdivi-1 has activity in preventing mitochondrial fission, we examined the effect of this drug of mitochondrial size in RK13 cells (Smith & Gallo, 2017), with increased length of mitochondria coming to a peak after 2 h treatment (Fig S12).

**Figure 8.**
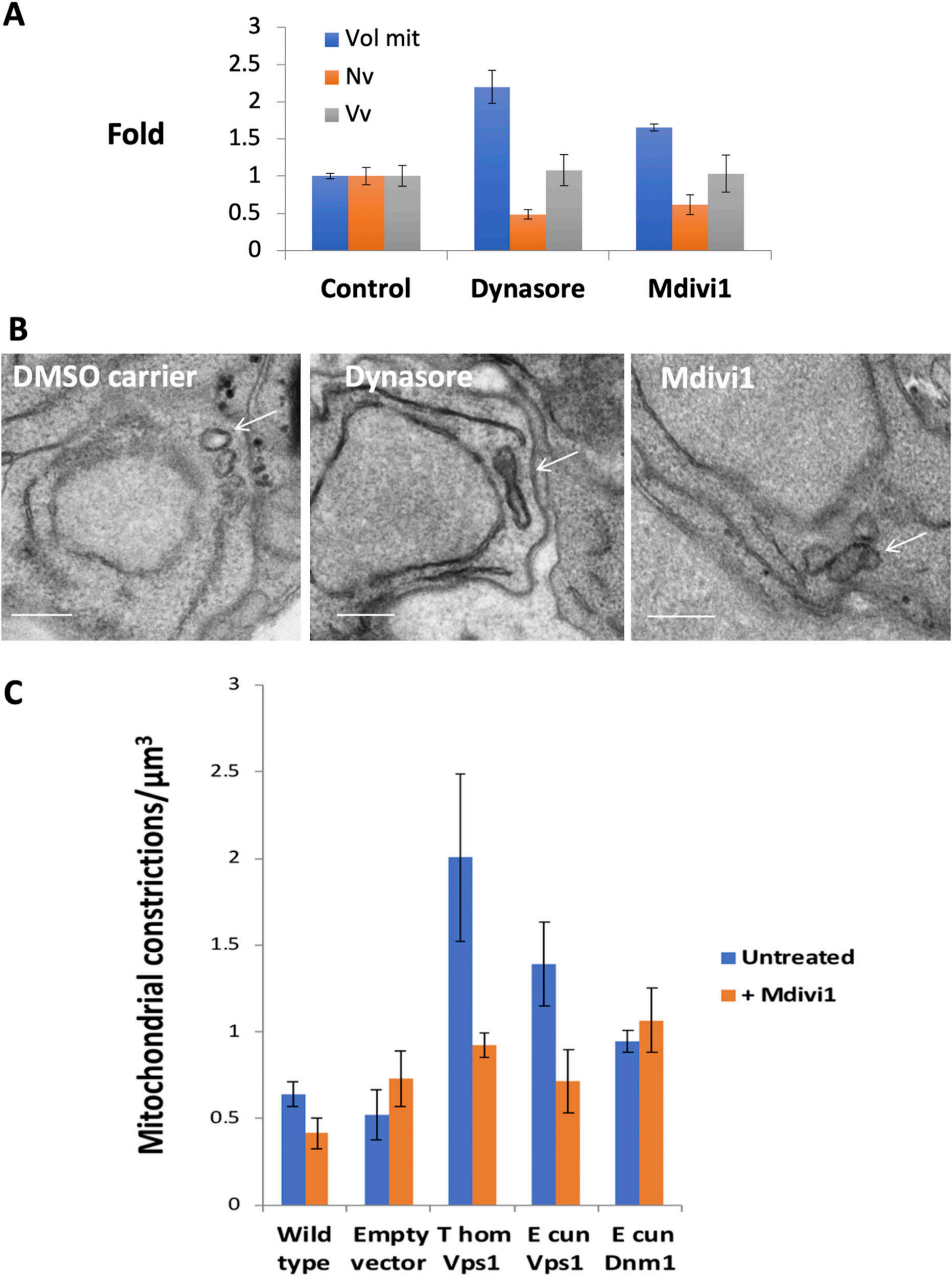
Dynamin inhibition inhibits mitosome scission and dynamin expression rescues constriction in *S pombe* dynamin deletion. **(A)** Dynamin inhibitors induce increase in mitosome size and reduction in numbers in *E*. *cuniculi* meronts. Mitosome volume (Vol mit) and number density (Nv) and volume density (Vv) were determined using stereological analysis using limited arrays of serial sections randomly located in the cells (n = 3; error bars SD). **(B)** In cells treated with Dynasore TEM reveals mitosomes (arrows) become elongated and display electron densities around the central waist portion. Mdivi1 induces mitosome enlargement (arrows), but the profiles are more globular in form. Bars, 200 nm. **(C)** Stereological quantification of mitochondrial constrictions in *S*. *pombe* in which Drp1 was deleted. The Vps1-like constructs from *T*. *hominis* (T hom) and *E*. *cuniculi* (E cun) increase the number of constriction sites per unit volume of cytoplasm, whereas the Dnm1 homologue does not.

RK13 cultures infected with *E*. *cuniculi* or *T*. *hominis* were treated for 2 h with either dynasore or Mdivi-1. After treatment of cells, the numbers and sizes of mitosomes were assessed using 3D quantitative electron microscopy (*E*. *cuniculi*; Fig 8). The data revealed that the numbers of mitosomes dropped, and the size increased after treatment with the inhibitors. We conclude that dynamin inhibitors inhibit scission leading to increased sizes of mitosomes over a 2-h period. Interestingly, treatment with dynasore increased the length of the mitosome, whereas mitosomes after Mdivi-1 treatment became more spherical in form (Fig 8).

In view of the reported off-target effects of dynamin inhibitors (Preta et al, 2015; Manczak et al, 2019) and to further test the hypothesis that the dynamin homologues were functional, we transformed plasmids expressing microsporidian dynamin-like proteins into *S*. *pombe* from which the *dnm1* gene had been deleted. Electron microscopy was used to check the activity of Mdivi-1 in WT *S*. *pombe* using stereologically estimated mitochondrial volume. This showed that the “star” volume of mitochondria (see the Materials and Methods section; [Gundersen & Jensen, 1985]) increased after 2 h of Mdivi-1 treatment to levels that were comparable with those in the *S*. *pombe dnm1∆* cells which exhibited extensive interconnected mitochondria (WT volume 0.14 µm^3^ [CE 22%]; WT + Mdivi-1 0.29 µm^3^ [CE 28%] and *dnm1∆* 0.26 [CE 7.7%]; N = 3 in each case). Next, these cells were transformed with full-length dynamin homologues and the effects on the mitochondrial morphology were assessed using quantitative EM, which involved counting the density of mitochondrial constrictions per unit volume of cytoplasm (Fig 8C). Whereas there was no increase in the number of isolated mitochondrial per se, there was an increased numerical density of mitochondrial constrictions within the cytoplasm after transformation with a plasmid expressing the Vps1-like protein but not with a plasmid expressing the Dnm1-like protein. Importantly, the increases in constriction densities were abolished when the yeast cells were treated with the Mdivi-1 inhibitor and were not seen after treatment with the empty plasmid. These data provided evidence for the mitochondrial constriction activity in microsporidian Vps-1-like dynamin.

## Discussion

### Mitosome structure in 3D

Our quantitative EM results reveal detailed features of microsporidian mitosome structure and life cycle. In ET 3D-reconstructions *E*. *cuniculi* mitosomes appear dumbbell-shaped and double-membraned, but lack canonical inner/outer membrane contacts, cristae or substructures within the matrix. The structural simplicity of this remnant mitochondrion reflects extreme genetic reduction whereby the archetypal multifunctional eukaryote mitochondrion, with ∼1,000 proteins, is reduced to the mitosome with a single conserved function in iron sulphur cluster assembly (Freibert et al, 2017), and an additional role in alternative respiration in some microsporidia including *T. hominis* (Sendra et al, 2022). The degree of simplification correlates well with our estimates of fractional volume, with mitosomes occupying 0.47% of the cytoplasm which is >10fold less than in animal cells (10–11% [Posakony et al, 1977]) or yeast (4–10%; [Damsky, 1976]). One canonical property of mitochondria was association of mitosomes with elements of endoplasmic reticulum. Proximity of the two organelles might suggest a functional association providing metabolic pathways such as using ER contacts such as cardiolipin synthesis (Tian et al, 2012) or phosphatidyl serine decarboxylation, although the latter appears to be absent in microsporidians. The ER might also contribute to mitochondrial division as it does in animal cells; however, we could not detect wrapping of ER cisternae around the waist of larger mitosomes (Friedman et al, 2011).

### Mitosome growth and division

Using a combined stereological, serial sectioning, and electron tomography approach during cell growth of *E*. *cuniculi*, we found the aggregate size of the mitosome compartment increases incrementally in concert with cell/nuclear growth, indicating a lack of cell cycle regulation which suggests that mitosomes provide a rather continuous supply of iron sulphur clusters (Freibert et al, 2017). As yet uncharacterised feedback processes are likely to balance the quantity of mitosome machinery with processes that depend on their function.

Our data also provide insight into the pattern and mode of mitosome division. In *E*. *cuniculi*, the gradual increase in aggregate volume is accompanied by graded increases in mitosome number, without a detectable step change. This indicates that mitosomal division continues during growth of the cell and mitosome mass. This scenario is supported by dynamin inhibitor studies, which showed a doubling of mitosome size inside a 2-h period. In contrast, strong cell cycle regulation of mitosome numbers has been inferred in studies on *Giardia* (Voleman et al, 2017; Tůmová et al, 2021), and mammalian cells, where numbers increase as cells prepare for stochastic inheritance during mitosis. Taken together, it appears that microsporidian mitosome division is unlikely to be under control of cell cycle/mitotic regulators and appears not to be timed to coincide with cell division.

### mSPB and its interaction with mitosomes

We observed a close association of mitosomes with mSPBs in both microsporidian species we studied. In *E*. *cuniculi*, most of the mitosomes remain closely associated with the mSPB throughout cell growth/cycle. Mitosomes contacted the mSPB at their curved ends and extended from the contact area as they grew, suggesting specialised regions of association that could involve direct association of mitosome and mSPB proteins. Interestingly, in *E*. *cuniculi*, the association was unaffected by microtubule depolymerising drugs which distinguish microsporidian mitosome from mitochondria of fission yeast where microtubule-dependent association with the SPB appears to depend on interaction with the ends of growing microtubules (Yaffe et al, 2003) and yeast CLASP (Chiron et al, 2008) rather than microtubule-associated motors.

In *E*. *cuniculi*, the low numbers of mitosomes, combined with consistent and even association with mSPBs in interphase and mitosis, suggest an ordered inheritance mechanism (Warren & Wickner, 1996). By this mechanism, attachment to the centrosome would serve to reliably maintain mitosomes for both daughter cells in contrast to random assortment which could generate daughters lacking mitosomes (and therefore, essential iron sulphur cluster assembly). Indeed, SPBs and therefore cells lacking associated mitosomes were not observed in our sample indicating reliable inheritance. Balanced partitioning could be further assisted by the even distribution of mitosome number across the two mSPBs we observed.

**Figure 9.**
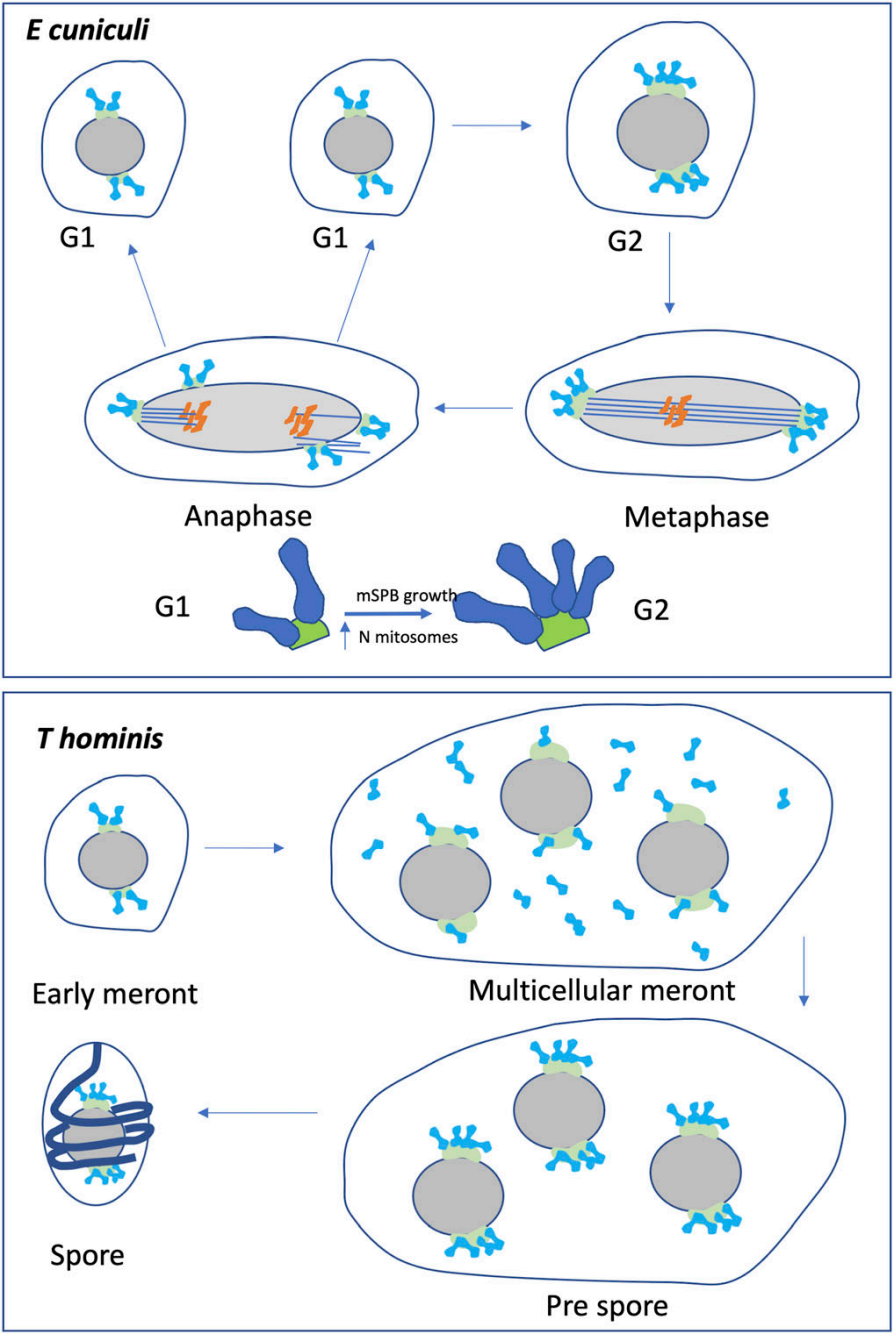
Models of mitosome biogenesis and inheritance. In both organisms, association with the microsporidian spindle pole bodies (mSPB) is proposed as a key factor in regulating inheritance. In *E cuniculi*, mitosomes remain associated with the mSPB throughout the cell cycle. In G1 each mSPB accommodates 1 or 3 mitosomes which divide and grow to produce 4–5 mitosomes on each mSPB, late in the cell cycle. The association of mitosomes with the mSPB is maintained during mitosis when each mSPB undergoes budding to form two newly formed mSPBs with their attached mitosomes (∼4–5) distributed across the resulting mSPBs. In *T hominis*, the mitosome number is greater and, although mitosomes are linked to the mSPB during early development, they become dissociated during multicellular stages. At the prespore stage and before cytoplasmic division of the syncytium, mitosomes once again associate with the mSPB ensuring proportionate inheritance during spore formation. See text for further details.

Future correlative light and electron microscopy studies could uncover the details of mitosome genesis and partitioning during cell and SPB duplication/division. In *T*. *hominis*, where the number of mitosomes is greater, the association with the mSPB appears reversible, with dispersed mitosomes clustering before cellularisation after a multinucleate stage (cellularisation) (Fig 9). In this case, the mitosome–mSPB binding may respond to cell cycle cues or events such as mSPB division. A recent study of *Giardia* described a scenario where a subset of mitosomes undergo apparent tethering and ordered inheritance at the flagellar basal body, whereas the more numerous peripheral mitosomes appear to be distributed to daughter cells stochastically (Tůmová et al, 2021). In that study, central mitosomes were connected to the flagellar basal body assembly via a microtubule-like structure that developed during mitosis. Interestingly, the stages of the *T. hominis* infection where we observed mitosomal clustering at the mSPB seemed to match those identified as sensitive to the treatment with an inhibitor of mitosomal alternative oxidase (AOX) (Sendra et al, 2022). This suggests that the changes in distribution of *T. hominis* mitosomes can potentially be synchronised with the proposed life cycle stage-specific switch in its metabolic functions (Sendra et al, 2022). Further studies to identify possible tethering mechanisms in microporidians will now be of interest.

Our investigation detected only four SPB protein homologues TUB4, SPC97, SPC98, and the nuclear membrane protein MPS, which suggests they can play important and conserved functions in microsporidia. The SPB of fission yeast undergoes a complex cycle of synthesis and division during the cell cycle (Rüthnick & Schiebel, 2016). During early interphase, a bridge forms on one side of each of the two SPBs from which a new SPB bud forms during G1. The process is complete during interphase and scission to form two SPBs each with a half bridge likely occurs during mitosis (Rüthnick & Schiebel, 2016). However, the process is still poorly defined partly because the structure is difficult to discern by electron microscopy. In the microsporidians studied here, we have not been able to identify a bridge or bud structures and a careful review of all serial section EM data and fluorescence localization of gamma- and alpha-tubulin revealed that only two mSPBs were found per interphase cell. It seems likely that mSPB separation of a preassembled, duplicated, mSPB is a rapid event that occurs during or immediately before telophase/early G1. Mitosome partitioning at mSPB could then confer reliable association of a mitosome(s), producing the observed even distribution of mitosome numbers across the mSPBs. Thus, we suggest that as *E*. *cuniculi* enter mitosis, the two (or more) mitosomes associated with each mSPB are evenly partitioned between the mSPBs as it divides, which is supported by finding of no increased inequality on the two mSPB in smaller (early postmitotic) cells. Such a mechanism is therefore an extremely reliable example of ordered inheritance. Further correlative light and electron microscopy studies will be required to resolve this issue. The model presented in Fig 9 is based on the quantitative data on mitosome growth, division, and association with mSPB as discussed above.

### Conserved dynamins function in mitosome scission

Mitochondrial dynamins are central to the control of mitochondrial fission in mammalian systems where there is a clear indication for cell cycle-driven scission (Kashatus et al, 2011). Two DRP homologues were identified in most of the microsporidian genomes analysed. Phylogenetic analysis provided strong evidence that the more conserved microsporidian DRP is a Vps1p orthologue (VPS). The origin of the second microsporidian DRP is less clear and could be a result of divergence from Dnm1p, Mx1-like, or Mgm1, or ancestral duplication of Vps1p. Each of the three sequenced *Nematocida parisii* isolates (Cuomo et al, 2012) contain only one VPS. *Nematocida* homologues were the most divergent of VPSs as they grouped together on a long branch at the base of other VPS orthologues, which could potentially reflect an adaptation associated with the ancestral loss of their second DRP. DRP homologues were not found in the most divergent microsporidian lineage, including species with a degraded glycolytic pathway (Wiredu Boakye et al, 2017). It is unclear how these extremely reduced Eukaryotes accomplish membrane vesicle constriction required for multiple cellular processes including that of mitochondrial fission. A more complex DRP repertoire was identified in representatives of two taxa most closely related to microsporidia, namely fungi and Cryptomycota (James & Berbee, 2012; James et al, 2013), suggesting that the reduced microsporidian DRP repertoire is a result of reductive evolution. Importantly, the two dynamins that rescued the extent of the constriction process were VPS orthologues.

Protein sequence comparison on the primary and secondary structure levels and Alphafold2 structure predictions revealed conservation of Dynamin fold with GTPase, MD and GED domains and potential loss or at least high reduction of the region corresponding to the Insert B in mammalian Drp1 and yeast Dnm1p from microsporidian DRPs. In *S. cerevisiae*, Dnm1p Insert B interacts with an N-terminal WD40 domain of an adaptor proteins Mdv1p (Tieu et al, 2002; Bui et al, 2012). The C-terminal region of the adaptor protein interacts with the outer mitochondrial membrane anchor protein Fis1p (Mozdy et al, 2000; Tieu et al, 2002). The results of BlastP and HMMER searches together with phylogenetic analysis suggest that microsporidian homologues of proposed interaction partners involved in recruitment of Dnm1p/Drp1 to mitochondria in yeast (Griffin et al, 2005; Cerveny et al, 2007; Hammermeister et al, 2010; Lackner et al, 2013) including Mdv1p (Tieu et al, 2002) and Fis1p (Mozdy et al, 2000) or mammals (Palmer et al, 2011) might have been lost in microsporidia or might be too divergent to be identified. In addition, the sequence motif present in Dnm1p that is proposed to be responsible for interaction with Mdv1p in *S. cerevisiae* (Bui et al, 2012) appears to be lost together with most of the microsporidian DRPs region corresponding to yeast Insert B.

Our data suggest progressive increases in mitosome number during interphase in *E*. *cuniculi* are dependent on dynamin function. First of all, treatment of infected cultures with inhibitors of dynamin (Macia et al, 2006; Cassidy-Stone et al, 2008) decreased mitosome number and increased their size during 2-h treatments as measured using EM-based stereology. The mitochondrial constriction function in yeast (Cassidy-Stone et al, 2008) is sensitive to Mdivi-1, which inhibits the assembly of Drp1 and GTPase Drp1 enzymatic activity in vitro while not inhibiting mitochondrial fusion. Mdivi-1 binds outside the GTPase domain that is involved in oligomeric assembly, thereby inhibiting GTPase activity. The other inhibitor dynasore is a selective noncompetitive inhibitor of the protein dynamin, which has activity against the VPS-like dynamin. Because dynamin inhibitors can have off-target effects, we also carried out experiments in which rescue of scission-related mitochondrial constriction was assessed in dynamin-deficient fission yeast expressing microsporidian dynamin. Here, we found that the two VPS dynamins encoded by the microsporidian genomes were active in rescuing the mitochondrial constriction process—supporting their role in scission related mechanisms. Interestingly, although mitochondrial dynamins are known to work in concert with accessory proteins, we could not find evidence for their existence in microsporidians. One possibility is that the rapid and well-known evolutionary divergence in microsporidians may make these accessory proteins undetectable by sequence analysis or there maybe novel mechanisms of scission involving dynamin in these organisms. More detailed studies are now required to identify the pathways of mitosome scission.

## Materials and Methods

### Antibodies and cells

RK cells (RK13) were grown in 5% CO_2_ 95% air at 35°C in MEM containing GlutaMAX supplemented with 10% heat-inactivated foetal calf serum, kanamycin 100 μg ml^−1^, penicillin 100 μg ml^−1^, streptomycin 100 μg ml^−1^, and Fungizone 1 μg ml^−1^ and were infected with *E*. *cuniculi* or *T. hominis*.

Full-length *T. hominis* mtHSP70, *T. hominis* γ-tubulin (*Th*TUB4, orf_785; GenBank: ELQ76224.1), and *E. cuniculi* γ-tubulin (*Ec*TUB4; GenBank: NP_597196.1) were cloned into a pET16 expression plasmid and expressed in the *E. coli* BL21 (DE3) as His-tagged recombinant proteins. *T. hominis* MPS3 coding gene (orf_2670; GenBank: ELQ74436.1), excluding the nucleotide sequence region (472–522) corresponding to a transmembrane domain, and a 3′ hairpin region (1,063–1,107) was cloned into pQE40 expression plasmid and expressed in the *E. coli* M15 as a His-tagged DHFR fusion protein. *T. hominis* actin coding gene (orf_1377; GenBank: ELQ75705) excluding nucleotide sequence fragments (127–567, 907–1,134) sharing high-sequence homology with the mammalian host actin was cloned into pQE40 expression plasmid and expressed in the *E. coli* M15 as a His-tagged DHFR fusion protein. Expressed proteins were purified using the BugBuster reagent, following the manufacturer’s protocol with the addition of Benzonase and lysozyme. The purified proteins were separated by SDS–PAGE, and bands containing 1 mg protein were cut out from the polyacrylamide gels and used for the commercial (Agrisera) generation of rabbit (*Ec*TUB4 and *Th*TUB4), and rat (*Th*MPS3; ThActin) polyclonal antisera. Each of the animals was immunized three times (weeks 1, 5, and 9) followed by the ELISA test (week 11), the final immunization (week 13), and the final bleed (50–70 ml rabbit; 3–4 ml rat; week 15).

Antibodies against *Ec*TUB4, *Th*TUB4, and *Ec*mtHSP70 were affinity purified by incubating nitrocellulose membranes containing ∼500 μg of purified proteins with 5 ml of antisera diluted 1:10 in mTBST (TBS; 0.1% Tween 20; 5% milk) overnight at 4°C with rocking followed by three TBST and one TBS wash. The purified antibodies were eluted from the membrane with 0.2 M glycine-HCl, pH 2.5 for 10 min, neutralised to pH 7.0 with unbuffered 1 M TRIS and NaCl (final concentration of 50 mM), and concentrated to 1 mg/ml (measured with NanoDrop, Thermo Fisher Scientific). The buffer was changed for PBS, using protein concentrator ultrafiltration centrifugal tubes (100,000 MWCO; Thermo Fisher Scientific), and antibodies were stored at −20°C, and used at 5 μg/ml for IF, or 1 μg/ml for Western blotting. The affinity purified EcmtHSP70 antibodies were directly conjugated with a fluorescent dye (488; Alexa Fluor) using APEX Antibody Labeling Kit (A10495; Invitrogen) following the manufacturer’s protocol. Antibodies against *E. cuniculi* HSP70 were prepared as described in Goldberg et al (2008).

### Electron microscopy

Infected monolayers were fixed in 0.5% glutaraldehyde in 0.2 M PIPES (buffer; pH 7.2; 15 min at RT) and scraped from the culture dish, before being resuspended in 1 ml of fixative and sedimented for 15 min at 16,000*g* in a plastic centrifuge tube. Pellets were washed three times in buffer (5 min per wash).

For structural analysis, pellets were post fixed and embedded in epoxy resin (Araldite; Agar Scientific) as described in Hacker et al (2014). For cryomicrotomy, the cell pellet was cryoprotected in 2.3 M sucrose in PBS (overnight at 4°C) and small fragments mounted on specimen carriers and plunge-frozen in liquid nitrogen before preparations of 80-nm-thick cryo-sections at −100°C (EM FC7 ultracryomicrotome; Leica). Ultrathin sections were picked up and thawed on 2.1 M sucrose/2% wt/vol methylcellulose (pre-mixed in equal volumes) before mounting on carbon/pioloform-coated EM copper grids (Agar Scientific). For immuno-gold labelling, grids were washed first in deionized water (three 5-min washes at 0°C) and then in PBS (single wash at RT). After an initial blocking step on 0.5% fish skin gelatin (Sigma-Aldrich) in PBS, the grids were labelled using rabbit antisera raised against Hsp70p followed by 10 nm protein-A gold (BBI Solutions). After washes in PBS and deionized water the sections were floated on droplets of 2% wt/vol methylcellulose/3% wt/vol uranyl acetate (mixed 9:1) before air drying (as described in Griffiths et al [1984]). To assess mitosome size, immunogold-labelled sections and epoxy-embedded sections were imaged with a JEOL 1200 EX transmission electron microscope operated at 80 keV and imaged using a GATAN Orius 200 digital camera (GATAN; Abingdon). Mitosomes were sampled systematic uniform random SUR (Lucocq, 2012); at a nominal magnification of 50,000x. Tiff format image files were imported in Adobe Photoshop CS6 and axes of gold labelled double-membraned structures (cryosections) or double-membraned structures (epoxy) were measured using the measuring tool. mSPBs were identified in epoxy resin-embedded samples as ribosome-free areas close to the nucleus (often surrounded by or connected to double-membraned mitosome structures; Fig S5D).

Electron tomography was performed on a 200-nm section contrasted with uranyl acetate and lead citrate using a JEOL 2200FS operated at 200 kV. Fiducial markers (10 nm colloidal gold) were applied to top and bottom of the sections. Tilt series were taken at 10 increments from −60° to +60° and tomograms prepared using iMOD software (Kremer et al, 1996). Segmentation was done manually and 3D models reassembled using AMIRA.

For quantitative analysis, serial sections were prepared from an epoxy resin-embedded material. 30–40 sections were prepared and meronts were identified in central sections by the criteria defined below. Micrographs of all identifiable mitosome profiles were taken (consecutive sections) and nuclei and cytoplasm profiles recorded on one in every five sections (average section thickness 43 nm as measured by the methods of Small (1968). Volumes of structures were estimated using Cavalieri’s method (Gundersen et al, 1999) using point counting as illustrated in Fig S4B and D. The volume data from conventional sections are liable to overprojection effects which lead to an overestimation. To correct for this effect, we estimated the volume of groups of mitosomes using both ET and conventional sections. The Cavalieri estimates showed that conventional ultrathin sections overestimated volume by a factor of 1.6-fold; a value, agreeing well with the error predicted using a model-based approach in which mitosomes were considered to be cylindrical (Weibel & Paumgartner, 1978; Fig S4B–D). This correction factor was applied to all estimates of mitosome volume as determined using conventional serial section TEM. To count mitosomes, a continuous series of adjacent serial sections (43 nm) were used. Scanning through the serial section images, mitosome profiles that were present in one section but not present in the next adjacent section were counted (Sterio, 1984), and again, the reliability of this procedure was confirmed using the thinner ET slices.

Vegetative stages of *T. hominis* (meronts) could be identified as single or multinucleated cell profiles situated within parasite vacuoles of RK13 cells. Sporulating parasites (sporonts, sporoblasts or spores) were distinguished by the presence of a discernible cell wall and/or the formation of the polar tube. Cytoplasm was defined as any area enclosed by the plasma membrane excluding nuclear profiles. The polar tube and associated structures (lamellar polaroplast) were not included in the quantification. Nuclear profiles were defined by nucleoplasm bounded by and including the nuclear envelope. Mitosomes were identified as double membrane-bound organelles measuring between 47–119 and 78–267 nm for minor and major axes, respectively, as defined using immunolabelling for Hsp70 and detailed in Fig 1. To investigate the role of microtubules in mitosome distribution, cells were treated for with either 10 µg ml^−1^ Nocodazole (4 h; Sigma-Aldrich) or 10 ng ml^−1^ Albendazole (3 h; Sigma-Aldrich) or in equal dilutions of DMSO carrier as controls. Dynamin inhibitors were applied to RK13 cells infected with microsporidians as detailed for immunofluorescence experiments.

*S. pombe* was processed for electron microscopy as described by Wright (Wright, 2000) using fixation in KMnO_4_ and embedded as described for RK13 cells. Ribbons of ultrathin sections were mounted on pioloform-coated EM copper slot grids (2 mm × 1 mm) and stained with Reynold’s lead citrate. Micrographs of randomly selected yeast cells at x 0.75–3 K and mitochondria (double membrane-bound profiles with evident cristae) at x 4–10 K magnification, respectively, were acquired using systematic sampling using a JEOL 1200 EX II TEM at 80 kV. Images were overlaid with a square lattice grid and star volume (Gundersen & Jensen, 1985) estimated for all sampled mitochondrial structures.

### Localisation of mSPB proteins and mitosomes using immunofluorescence/SIM

Confluent *T. hominis*-infected rabbit kidney cells were grown on glass coverslips and incubated for 2 h with either control (1:1,000; DMSO) or treatment (Mdivi-1 [50 μM] or Dynasore [80 μM] diluted from 80 mM stock dissolved in DMSO in serum-free DMEM) in a six-well plate. Cells were fixed in 50:50 acetone:methanol (vol/vol %) at −20°C for 2 h and washed thrice with PBS. Blocking was performed by incubating cells in PBS-containing FSG (0.5%; Sigma-Aldrich) for 1 h followed by three washes with PBS before incubating for 1 h with 1:250 dilution of *T. hominis* mitochondrial Hsp70 (ThmtHsp70). The coverslips were further incubated with goat anti-rabbit secondary antibody conjugated to fluorescent dye Alexa-fluor 594 (1:500; Molecular Probes) for 40 min in the dark and then treated with DAPI (1:000). Glass slides were incubated overnight and sealed with varnish for data acquisition. Two independent experiments were performed (n = 50, where n represents the total number of parasitic nuclei observed in each experimental condition). Under x100 magnification oil immersion lens, *T. hominis*-infected RK cells were randomly selected for deconvolution into stacks of multiple optical sections (1,024 × 1,024 pixels) after setting the cell thickness (upper limit: first optical section just before mitosomes were visible, and lower limit: after the section where mitosomes were no longer visible). Numbers of sections were highly variable across the cell sample population. Video files (.dv format) of cells were acquired using a Deltavision OMX system (Applied Precision) to generate optical sections with 3-D SIM spatial resolution. Cells in experiments using BrdU were imaged using a Nikon SIM, kindly provided by Professor Frank Gunn-Moore (University of St Andrews).

To facilitate the counting of labelled mitosomes, a lattice grid was placed on the section stack in softWoRx program (Softworx). All the visible mitosomes surrounding a parasitic nucleus were systematically counted in every square, starting from the second optical section (first one was used as a reference frame and therefore excluded during counting). Every “new” mitosome profile appearing in the underlying optical sections were added to the cumulative mitosome count of the previous section. To nullify repeated measurement, only those mitosome profiles were included that were completely inside a square within the grid lattice. For the cells on the borders of each square, profiles visible on the top and left-hand side borders were included.

RK13 cells infected with *T. hominis* or *E. cuniculi* grown on coverslips were fixed in 50:50 vol/vol methanol/acetone at −20°C for 10 min. After blocking with 5% (wt/vol) milk in PBS (mPBS), slides were incubated overnight with a mPBS solution containing the relevant affinity-purified antibodies or antisera at 4°C, washed three times in PBS, and then incubated for 1 h with the secondary goat anti-rat or anti-rabbit antibodies (Thermo Fisher Scientific) conjugated to Alexa Fluor 594 (red) or 488 (green). For the double labelling experiments, slides were washed with PBS and the same labelling protocol was followed for the second set of antibodies. After the antibody labelling, cells were incubated with DAPI in PBS for 5 min to visualize the host and the parasite nuclei. Slides were mounted using ProLong Gold (Thermo Fisher Scientific), and sealed using CoverGrip coverslip sealant (Biotium). Cells were visualized using either a Nikon A1R confocal microscope with x63 oil lens, a Zeiss Axioimager II microscope with a x100 phase contrast oil lens, or with a super-resolution stimulated emission depletion Leica SP8 STED with x100 STED oil lens. The images were processed in FIJI (Schindelin et al, 2012) or in VoloCity (PerkinElmer). Fluorescent point signals were automatically detected and quantified using “Find Spots” function implemented in VoloCity. Volumetric fluorescent objects were automatically detected using “find objects” function (minimum object size 0.01 μm^3^) and objects that were at least partially overlapping were detected using “exclude non-touching” function implemented in VoloCity. Image deconvolution was performed in VoloCity with experimentally acquired point spread functions using 0.1 μm TetraSpeck Microspheres (T7279; Thermo Fisher Scientific) on slides prepared the same way as those of the antibody-labelled samples.

### Synchronised time course of *T. hominis* infection

The fresh spores were isolated on the day of the experiments by lysing 175 cm^2^ culture flask of RK13 cells heavily infected with *T. hominis* in 0.2% triton X-100 PBS, followed by sonication (3 s × 45 s on ice), and Percol purification of the microsporidian spores. A single flask of heavily infected RK culture was required for the infection of a single 24-well plate. To initiate the synchronised time course of the microsporidian infection, the Percol-purified spores of *T. hominis* (5–20 × 10^6^/cm^2^) were added to the 80% confluent RK13 monolayer, and incubated for 2 h at 37°C. The incubation of the spores was followed by a thorough washing (at least three washes) with DMEM to remove excess spores. For immunofluorescence microscopy, the ThRK13 cells were cultured in 24-well plates on round cover slips, and time-points were collected by fixing the cells in methanol/acetone followed by the IF.

### Sequence analysis

BlastP searches were performed using a local version of the Blast package 2.2.29+ (Altschul et al, 1990). Accession numbers of *S*. *cerevisiae* protein sequences and human protein sequences used as queries in BlastP searches are available on request. The same sequences were used as queries in Pfam-A and Pfam-B (Finn et al, 2014) database searches. HMM profiles downloaded from Pfam-A and HMM profiles generated with hmmbuild (HMMER3 (Eddy, 1998) using alignments downloaded from Pfam-B were used in HMM profile searches with hmmsearch HMMER3, (Eddy, 1998). BlastP and HMM searches were performed against local protein sequence databases containing microsporidian sequences *Nosema ceranae* BRL01, (Cornman et al, 2009), *Nosema bombycis* CQ1, *Nosema apis* BRL 01, (Chen et al, 2013); *E. cuniculi* GB-M1, (Katinka et al, 2001); *E. intestinalis* ATCC 50506, (Corradi et al, 2010); *Encephalitozoon hellem* ATCC 50504, (Corradi et al, 2010); *Encephalitozoon romaleae* SJ-2008, (Pombert et al, 2012); *Vittaforma corneae* ATCC 50505 (GCF_000231115.1), *Enterocytozoon bieneusi* H348, (Akiyoshi et al, 2009); *Edhazardia aedis* USNM 41457, *V. culicis floridensis* (http://www.broadinstitute.org/), *T. hominis*, (Heinz et al, 2012); *Spraguea lophii* 42_110, (Campbell et al, 2013); *N. parisii* ERTm1, (Cuomo et al, 2012); *N. parisii* ERTm3, (Cuomo et al, 2012); *Nematocida* sp1 ERTm2 (Cuomo et al, 2012), downloaded from GenBank (http://www.ncbi.nlm.nih.gov/). tBlastN (Altschul et al, 1990) searches were performed against microsporidian NCBI WGS (Whole Genome Shotgun) sequences. Protein sequences used in the construction of the dynamin phylogenetic trees were downloaded from GenBank and the JGI Genome Portal MycoCosm (Grigoriev et al, 2014) (http://genome.jgi.doe.gov/).

Multiple sequence alignments were generated using MUSCLE (Edgar, 2004) and trimmed using trimAL (Capella-Gutiérrez et al, 2009) using the automated1 or gappyout method. Maximum likelihood trees were generated with RaxML (Stamatakis et al, 2005) under models determined with ProtTest3 (Guindon & Gascuel, 2003; Darriba et al, 2011) and with 100 rapid bootstrap replicates. Bayesian analyses were performed using PhyloBayes (Lartillot et al, 2009) in four chains that were run in parallel until convergence as determined using the bpcomp and tracecomp programs of the PhyloBayes package (Lartillot et al, 2009). The fit of models used to construct Bayesian phylogenies was evaluated using posterior predictive analysis (Bollback, 2002) implemented in the ppred program of the PhyloBayes package (Lartillot et al, 2009). TMHMM was used for prediction of transmembrane helices (Sonnhammer et al, 1998; Krogh et al, 2001). Secondary structure predictions were generated using the Jpred 3 server (Cole et al, 2008) (www.compbio.dundee.ac.uk/jpred/). Available AlphaFold (Jumper et al, 2021; Varadi et al, 2022) structure predictions (UniProt IDs: Q8SSJ7, Q8SR00, and L7JSH7) were downloaded from (AlphaFold Protein Structure Database). The structures of *R. allomycis* MX1-like, *S. cerevisiae* Vps1p and Dnm1p, and *T. hominis* Drp, were predicted using ColabFold v1.5.2: AlphaFold2 using MMseqs2 online server (default mode) (Mirdita et al, 2022). The predicted protein structures were visualised in PyMOL (The PyMOL Molecular Graphics System, Version 2.0 Schrödinger, LLC). Formatting and analysis of multiple sequence alignments was performed in Jalview (Waterhouse et al, 2009), Seaview (Gouy et al, 2010), and ALINE (Bond et al, 2009). The pairwise percentage of sequence identity were calculated using the -output = sim function implemented in T-coffee.

### Expression of microsporidian Dmn1/Vps1 homologues in fission yeast

To analyse the function of the microsporidian Dmn1/Vps1 homologues in *S. pombe*, *E. cuniculi* ORFs *ECU01_1210* (*Dmn1*), and *ECU10_1700i* (*Vps1*) and *T. hominis* ORFs *THOM_1614* (*Dmn1*) and *THOM_2681* (*Vps1*) were amplified from genomic DNA prepared from purified *T. hominis* spores using the high-fidelity Q5 DNA polymerase (New England Biolabs), cloned into the fission yeast expression vector pREP3X, 3′ to the thiamine-repressible *nmt1* promoter (Maundrell, 1990), and sequenced. Details of the primers used for PCR amplification can be obtained from the authors by request.

**Figure S13.**
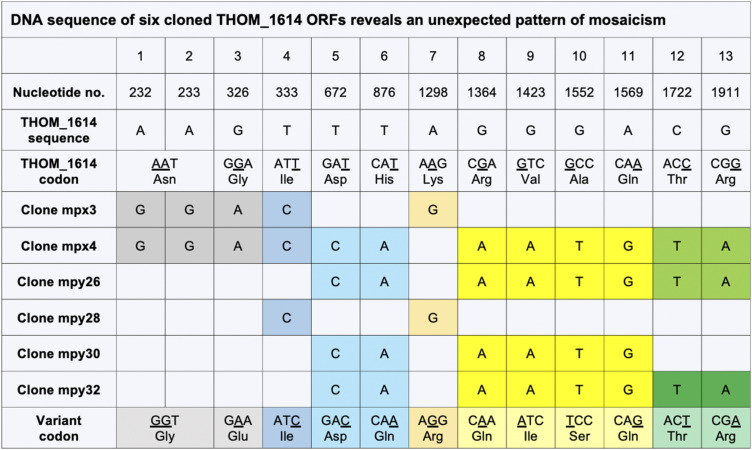
Sequencing of six independent clones of THOM_1614 reveals an intriguing pattern of mosaicism consistent with the presence of multiple THOM_1614 alleles within the population and extensive recombination between these alleles. Five of the six clones had different sequences, with none matching the published THOM_1614 ORF sequence (GenBank accession number JH993964.1).

In the case of THOM_1614 (Dmn1), sequencing of six independent clones revealed an intriguing pattern of mosaicism consistent with the presence of multiple THOM_1614 alleles within the population and extensive recombination between these (Fig S13). Five of the six clones had different sequences, with none matching the published THOM_1614 ORF sequence (GenBank accession number JH993964). Given the uncertainty over the nature of these clones, the function of this protein was not pursued further.

To examine the ability of the remaining Dmn1/Vps1 homologues to rescue loss of *dnm1* function in fission yeast, plasmids pREP3X, pREP3X-EcDmn1, pREP3X-EcVps1, and pREP3X-ThVps1 were transformed into *S. pombe* strain SP772 (*dnm1::kanMX4 leu1-32 ura4-D18 ade6*) and transformants obtained on EMM medium supplemented with uracil and adenine, with or without 5 μg/ml thiamine to repress or derepress the nmt1 promoter. SP772 (Kim et al, 2010) was a generous gift of Dr J. Hayles (Crick Institute, London).

To express the microsporidian Dmn1/Vps1 homologues as N-terminal GFP fusions in *S. pombe*, the ECU01_1210 (*Dmn1*), ECU10_1700i (*Vps1*), and THOM_2681 (*Vps1*) ORFs were amplified by PCR using the appropriate pREP3X plasmids described above as templates, cloned into plasmid pREP3X-GFP, and sequenced. Details of the primers used for PCR amplification can be obtained from the authors by request. Plasmid pREP3X-GFP (S.M., unpublished) is a modified form of pREP3X carrying the GFP (S65T) ORF downstream of the *nmt1* promoter, flanked by *Xho*I and *Sal*I sites (5′) and *Bam*HI and *Sma*I sites (3′). For subsequent analysis, plasmids pREP3X-GFP, pREP3X-GFP-EcVps1, and pREP3X-GFP-ThVps1 were transformed into *S. pombe* strains SP322 (*leu1-32 ura4-D18*) and SP775 (*dmn1:kanMX6 leu1-32 h*^*+*^) and transformants obtained as above. Strain SP775 (Jourdain et al, 2008, 2009) was kindly supplied by Dr Isabelle Jourdain (formerly at the University of Exeter, UK).

## Supplementary Material

Reviewer comments
